# Beyond ribose and phosphate: Selected nucleic acid modifications for structure–function investigations and therapeutic applications

**DOI:** 10.3762/bjoc.17.76

**Published:** 2021-04-28

**Authors:** Christopher Liczner, Kieran Duke, Gabrielle Juneau, Martin Egli, Christopher J Wilds

**Affiliations:** 1 Department of Chemistry and Biochemistry, Concordia University, Montréal, Québec H4B 1R6, Canada; 2 Department of Biochemistry, Vanderbilt Institute of Chemical Biology, and Center for Structural Biology, School of Medicine, Vanderbilt University, Nashville, Tennessee 37232, United States

**Keywords:** antisense, chemically modified oligonucleotides, crystallography, siRNA, structure

## Abstract

Over the past 25 years, the acceleration of achievements in the development of oligonucleotide-based therapeutics has resulted in numerous new drugs making it to the market for the treatment of various diseases. Oligonucleotides with alterations to their scaffold, prepared with modified nucleosides and solid-phase synthesis, have yielded molecules with interesting biophysical properties that bind to their targets and are tolerated by the cellular machinery to elicit a therapeutic outcome. Structural techniques, such as crystallography, have provided insights to rationalize numerous properties including binding affinity, nuclease stability, and trends observed in the gene silencing. In this review, we discuss the chemistry, biophysical, and structural properties of a number of chemically modified oligonucleotides that have been explored for gene silencing.

## Introduction

The natural nucleic acids sugar-phosphate backbone comes in two flavors, 2'-deoxyribose in DNA and ribose in RNA. However, this relative simplicity combined with the five natural bases, adenine (A), cytosine (C), guanine (G), thymine (T) and uracil (U, in RNA) belies the fact that both DNA and RNA are decorated with chemical modifications. For a catalogue of natural modifications in DNA, see https://dnamod.hoffmanlab.org/ [1], and in RNA, see https://iimcb.genesilico.pl/modomics/ [2]. In DNA, base modifications are much more common than those in the backbone and play a central role in epigenetics, such as, for example, the ‘fifth base’ 5-methylcytosine (5mC) [3]. In the backbone, chemical modification appears to be limited to the phosphorothioate *R*p-stereoisomer (*R*p-PS, i.e., phosphate with one of the non-bridging oxygens replaced by sulfur) in bacterial genomes, where it may serve a protective role against nucleases [4] and its loss results in genomic instability [5]. There are over a hundred known base modifications in RNA and the *R*p-PS backbone modification occurs in ribosomal RNA (rRNA) of both pro- and eukaryotes [6]. A very common natural modification that concerns the ribose moiety is 2'-*O*-methylation (2'-*O*Me). 2'-*O*Me nucleotides are scattered throughout all types of RNA, including mRNA, tRNA, rRNA, snRNA, snoRNA, miRNA and viral RNA [7–9]. Moreover, the modification occurs irrespective of the nature of the base and is therefore also referred to as Nm (N = A, C, G, 5mU, U, ψU, I, etc.) [10].

The specific role(s) an individual modification plays is often not known, but we can surmise involvements in transcription, translation, replication, splicing and other fundamental processes in biological information transfer. More specifically, they can affect chemical and thermodynamic stability, folding, secondary and tertiary structure, activity and interactions between nucleic acids, proteins and receptors. Particularly, as far as improving metabolic stability, pairing properties (RNA affinity), protein binding and transport/cellular uptake are concerned, chemical modifications are a prerequisite for the discovery and development of oligonucleotide therapeutics [11–15]. Thus, the natural PS and 2'-*O*Me backbone modifications provide improved resistance to degradation by exo- and endonucleases and they both affect protein binding [16–17]. Eight of the now approved 13 oligonucleotide drugs feature the PS modification in the backbone and all four approved siRNA therapeutics: ONPATTRO^®^ (patisiran, 2018), GIVLAARI^®^ (givosiran, 2019), OXLUMO^®^ (lumasiran, 2020) and LEQVIO^®^ (inclisiran, 2020) have 2'-*O*Me modifications [18–21] (https://www.oligotherapeutics.org/20th-anniversary-of-rna-interference-in-mammalian-cells/). Interestingly, both 2'-*O*Me [22] and PS [23] date back to the 1960s and constitute the earliest modifications reported by chemists along with the synthesis of 2'-deoxy-2'-fluoro-nucleosides (FRNA) [24].

The negatively charged phosphodiester linkages in the backbones of DNA and RNA are of fundamental importance for reactivity, stability, conformation and hydration [25–26]. The sugar moieties in DNA and RNA determine the shape of the double helix, i.e., the facile flip between the C2'-*endo* (B-form DNA) and C3'-*endo* (A-form DNA) puckers by deoxyribose and the shift toward the C3'-*endo* pucker due to the presence of the 2'-OH in RNA [27–28]. As well, the seemingly small difference of a single hydroxy group between the sugars in DNA and RNA is at the origin of the vastly expanded fold [29–32] and functional spaces of RNA [33–39]. Perhaps less known is the fact that the sugar moiety in the backbone of a nucleic acid determines the base pairing priorities. For example, in DNA G:C > A:T whereas in homo-DNA (2',3'-β-ᴅ-dideoxyglucopyranose nucleic acid) G:C > A:A ≈ G:G > A:T (reverse Hoogsteen A:A and G:G pairs) ([40] and cited references). Messenger RNA is the target of both the antisense and RNAi strategies to interfere with biological information transfer prior to production of proteins, enzymes and receptors that may be inhibited by small-molecule and antibody therapeutics. However, native RNA oligonucleotides do not possess sufficient metabolic stability for in vivo applications. Therefore, chemical modification is absolutely essential to re-engineer RNA into a therapeutic tool [15].

**Figure 1:**
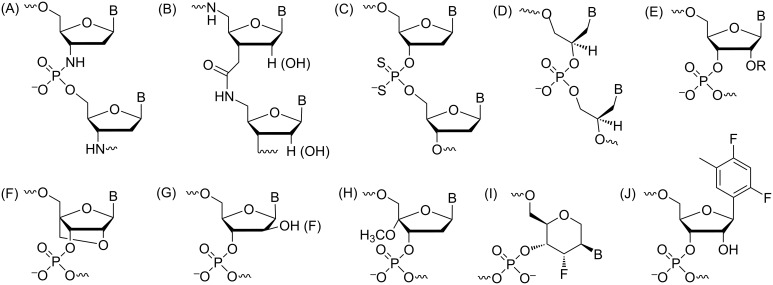
Structures of the chemically modified oligonucleotides (A) N3' → P5' phosphoramidate linkage, (B) amide (AM1) linkage, (C) phosphorodithioate (PS2), (D) glycol nucleic acid (*R*-isomer), (E) 2'-*O*-alkyl modifications (R = -CH_3_, -CH_2_CH_2_OCH_3_), (F) locked nucleic acids (LNA)/bridged nucleic acids (BNA), (G) arabinose (ANA) and arabinofluoro (FANA) nucleic acids, (H) C4'-modified nucleic acids, (I) 3'-fluorohexitol nucleic acid, (J) ribo-difluorotoluyl-modified nucleic acid.

The chemical make-up of RNA, i.e., the ribose-phosphate backbone, has inspired countless strategies to chemically modify either the sugar [12,41–44], or the phosphate (e.g., amide-RNA [45]), or both [46–47]. In addition, the ribose has been replaced with alternative sugar moieties, such as a tetrose (ʟ-α-threofuranose, TNA [48]), and hexoses (e.g., hexitol, HNA [49]; altritol AtNA [50]; xylol XyNA [51]), or cyclohexene (CeNA [52]), a morpholino moiety (PMO [53]), and an acyclic, chiral glycol linker (GNA [54]), to generate so-called xeno nucleic acids (XNAs [55–56]). In arguably the most radical alternative nucleic acid pairing system, peptide nucleic acid (PNA), the sugar-phosphate backbone is replaced by an amide-based, neutral and achiral scaffold that allows cross-pairing with both DNA and RNA as well as formation of double- and triple-stranded species [57]. Despite this growing universe of modifications, 2'-modifications, such as the original 2'-*O*Me, 2'-*O*-(2-methoxyethyl) (MOE [58–59]), and locked nucleic acid (LNA [44,60]) as well as the FRNA analogue [61–63] along with the phosphorothioates, will likely remain critical for the development of new oligonucleotide-based therapeutics. In the present review, we will summarize the properties of selected backbone modifications (Figure 1) and discuss investigations regarding their structure and function and, if applicable, their importance for therapeutic applications.

## Review

### Internucleotide linkage modifications

#### N3' → P5' phosphoramidate

**Scheme 1:**
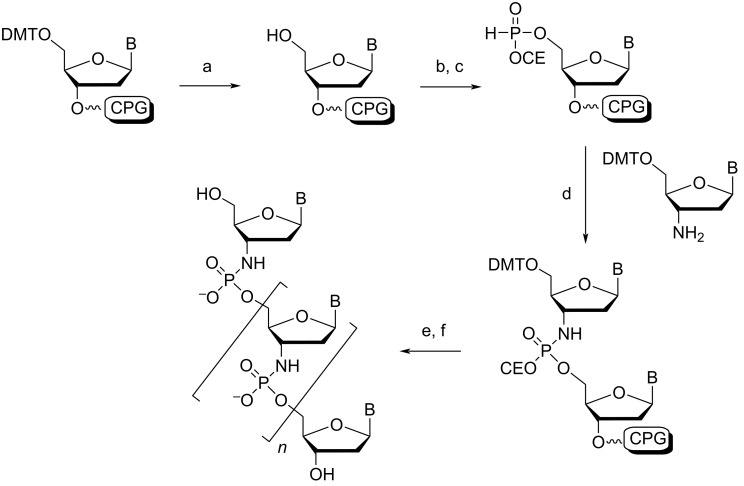
Synthesis of a N3' → P5' phosphoramidate linkage by solid-phase synthesis. (a) dichloroacetic acid; (b) ClP(NiPr_2_)(OCE); (c) tetrazole/water; (d) triethylamine/carbon tetrachloride; (e) repeat steps a–d; (f) detritylate then deprotect with NH_3_. DMT = dimethoxytrityl, CPG = succinyl-linked long chain alkylamine controlled pore glass solid support, CE = 2-cyanoethyl. Adapted from [64].

The N3' → P5' phosphoramidate DNA (3'-NP DNA) contains a negatively charged internucleotide linkage, but one of the bridging oxygens is replaced by a nitrogen (Figure 1A). The 3'-NP linkage is generated during solid-phase synthesis where the incoming protected 5'-DMT-3'-aminonucleoside couples to the 5'-H-phosphonate in the presence of a base (Scheme 1) [64].

In comparison with natural phosphodiester oligonucleotides, these modified oligonucleotides display improved nuclease resistance and an enhanced duplex thermal stability of 2.3–2.6 °C per linkage independent of nucleotide sequence and base composition [65]. The presence of alternating phosphodiester and phosphoramidate linkages within an oligonucleotide resulted in improved binding to RNA relative to DNA. Homopyrimidine 3'-NP DNA forms a stable triplex at neutral pH with double-stranded DNA and RNA [64–66].

These attributes, nuclease stability, and hybridization to single and double stranded nucleic acid targets have led to studies to investigate 3'-NP DNA for antisense and antigene purposes. For example, as an antisense agent in the treatment of human leukemia [67], as an inhibitor of transcription elongation targeted to proviral HIV DNA [68], and as a triplex-forming oligonucleotide that selectively binds a sequence within the chromatin structure of cell nuclei [69]. Remarkably, 3'-NP DNA can also act as an RNA mimic in interactions with binding proteins despite lacking a ribose moiety, making them useful nuclease-resistant probes for studying RNA–protein interactions [70].

**Figure 2:**
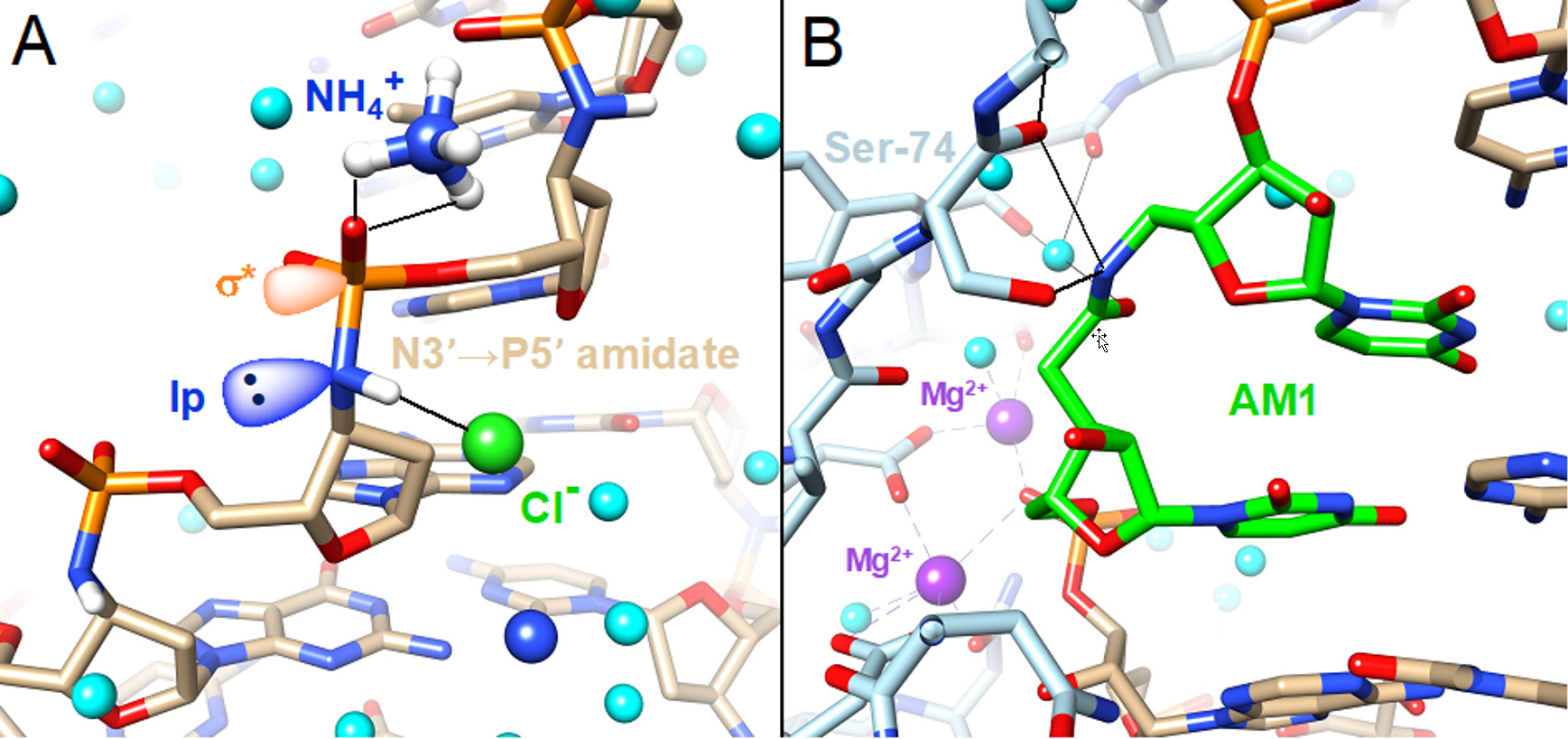
Crystal structures of (A) N3' → P5' phosphoramidate DNA (PDB ID 363D) [71] and (B) amide (AM1) RNA in complex with *Bacillus halodurans* RNase H (PDB ID 5VAJ) [73]. The relative orientation of the N3' n and P–O5' σ* orbitals in the backbone of 3'-NP DNA are consistent with an anomeric effect. The 3'-nitrogen is H-bonded to a chloride anion (green sphere) and the phosphate group forms a salt bridge to ammonium. Water molecules are cyan spheres and H-bonds are drawn with thin lines.

To better elucidate the structural features of 3'-NP DNA responsible for this enhanced selective binding and stability, the Egli group determined the crystal structure of the fully modified 3'-NP DNA duplex with the sequence 5'-d(CnpGnpCnpGnpAnpAnpTnpTnpCnpGnpCnpG)-3' at 2 Å resolution [71]. It was found that the overall duplex structure adopted by 3'-NP DNA resembles that of an RNA-like A-form double helix. The deoxyribose ring of phosphoramidate DNA is locked in a northern (C3'-*endo*) conformation due to the decreased gauche effect between 4'-O and the 3'-N compared to the 4'-O and 3'-O interactions in DNA. The 3'-amino moieties in the structure’s backbone were found to coordinate a larger amount of water molecules, on both the backbone and at groove sites. This increased hydration, as well as the configuration of the 3'-amino group enables the hydrogen atom to orient towards anions (chloride) in the vicinity and the 3'-nitrogen lone pair engages in a lp → σ* anomeric effect with the antibonding orbital from the adjacent P–O5' bond (Figure 2A). This conjugation is surmised to cause considerably increased rigidity of the phosphoramidate sugar-phosphate backbone relative to native phosphodiester oligomers. This N-type sugar puckering and increased hydration of the sugar phosphate backbone could also account for the triplex-favoring properties of this modification [72].

#### Amide

While many amide backbone oligonucleotide variants exist, the focus of this review will be on the AM1-type shown in Figure 1B, as this is the most studied and therapeutically promising modification of its class (a summary of other amide variations can be found elsewhere [74–75]). The strategy used to incorporate this modification into DNA or RNA has been to first synthesize the nucleoside dimer phosphoramidite with the appropriate amide linkage, which can then be introduced into the strand by solid-phase synthesis. These dimers are synthesized by using an amide coupling reagent to condense a 3'-carboxylic acid nucleoside with a 5'-amine nucleoside, where the necessary protecting groups are present on the nucleobase and sugar moieties [76–77].

Unlike the phosphodiester linkage of natural DNA, the AM1 modification is an example of a non-ionic backbone. The crystal structure of a 13-mer RNA duplex with a single central AM1 modification revealed that this modification is accommodated in an A'-form duplex [75]. Interestingly, an unconventional C–H···O hydrogen bond was observed between the amide’s carbonyl oxygen and the nearby uracil C6–H6. The thermal stability of this modified duplex was, however, quite similar to native RNA. Typically, there is a decrease of 0.2–0.8 °C in the thermal stability of RNA/DNA hybrid duplexes for each AM1 modification [11,78]. NMR structural studies have shown that the AM1 modification is well tolerated in an RNA duplex, with little effect on the global structure [79]. Furthermore, siRNA duplexes with amide modifications at the 3'-overhang region show enhanced endonuclease and 3'-exonuclease resistance [80]. Thus far, the AM1 modification has not found great success in antisense therapeutics, owing to RNAse H not recognizing a uniformly modified AM1-DNA:RNA heteroduplex. Recently, however, an 18-mer AM1-DNA gapmer was synthesized, with 4 AM1 linkages on each flank of the oligonucleotide [81]. Once bound to its RNA target, RNAse H was able to completely degrade the RNA in just 30 minutes, demonstrating the effectiveness of AM1 modifications in chimeric oligonucleotides for antisense therapeutics.

While this lack of charge was also believed to render AM1-RNA incompatible with siRNA therapeutic strategies, as there was crystallographic data [82] that showed the main interaction between the phosphates of the RNA duplex and the Ago2 protein is electrostatic in nature, this was, however, not the case, owing to the observed increase in silencing activity for AM1-modified siRNAs with amide linkages at specific sites [75]. Structural insight into this observation was obtained using the crystal structure of the complex between *Bacillus halodurans* RNase H and the r(GAC ACC UGA UAM1UC) - d(GAA TCA GGT GTC) hybrid duplex [73]. Compared to the native complex, conformational changes in the RNA and protein were only observed around the site of the AM1 modification. Not only was the amide an ideal structural mimic of phosphate, it also possessed stabilizing hydrogen bonds between the amide N–H and the main chain oxygen and side chain Oγ of S74 (Figure 2B), explaining their tolerance towards efficient recognition by Ago2. Interestingly, however, disfavoring stabilizing interactions with Ago2 through an amide backbone modification can be therapeutically beneficial when placed in the proper site. This was exemplified by a recent study that placed a single AM1 backbone modification between nucleotides 1 and 2 at the 5'-end of the siRNA passenger strand, whereby the off-target effects of that strand were abolished and the activity of the guide strand was restored [83].

#### Phosphorodithioate

**Scheme 2:**
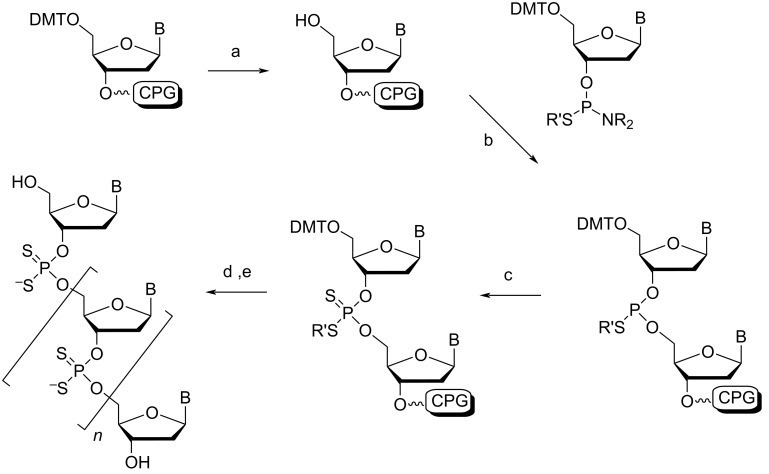
Synthesis of a phosphorodithioate linkage by solid-phase synthesis. (a) detritylation; (b) tetrazole; (c) sulfurization, capping, then washing; (d) repeat steps a–c; (e) detritylate then deprotect with NH_4_OH. R = pyrrolidino, R' = β-thiobenzoylethyl. Adapted from [85].

The synthesis of phosphorodithioate (PS2)-modified oligonucleotides was first described in 1991 by the Caruthers group [84]. Typically, each 2'-deoxynucleoside 3'-phosphorothioamidite is prepared by phosphitylating the protected nucleosides with tris(pyrrolidino)phosphine under tetrazole catalysis, followed by immediate treatment with monobenzoylethanedithiol. The 3'-phosphorothioamidites are incorporated into an oligonucleotide by standard solid-phase synthesis conditions, however, the oxidation step is replaced with sulfurization by elemental sulfur (Scheme 2) [85]. It should be noted that more efficient sulfurization agents exist with faster kinetics and higher solubility in organic solvents, useful for automated synthesis, such as the Beaucage reagent [86]. Conveniently, during deprotection of the support-bound oligonucleotide, aminolysis removes the β-thiobenzoylethyl group from the backbone to generate the free PS2-modified oligonucleotide. This modification is achiral at the phosphorus atom (Figure 1C), and thus, unlike the phosphoromonothioate (PS) analogues (extensively covered in other reviews [18,42,87–88]), the synthesized oligonucleotide is stereochemically pure. This simplifies their purification, as there is no longer the need to separate biochemically distinct diastereomers in order to make meaningful conclusions about the modification in a therapeutic or crystallographic context (although individual PS diastereoisomeric linkages can be resolved in electron density maps at sufficiently high resolution [18,89]). This modification has been attractive in antisense therapeutics as these altered oligonucleotides can form a hybrid duplex with unmodified RNA, which is recognized by RNase H [89–90].

**Figure 3:**
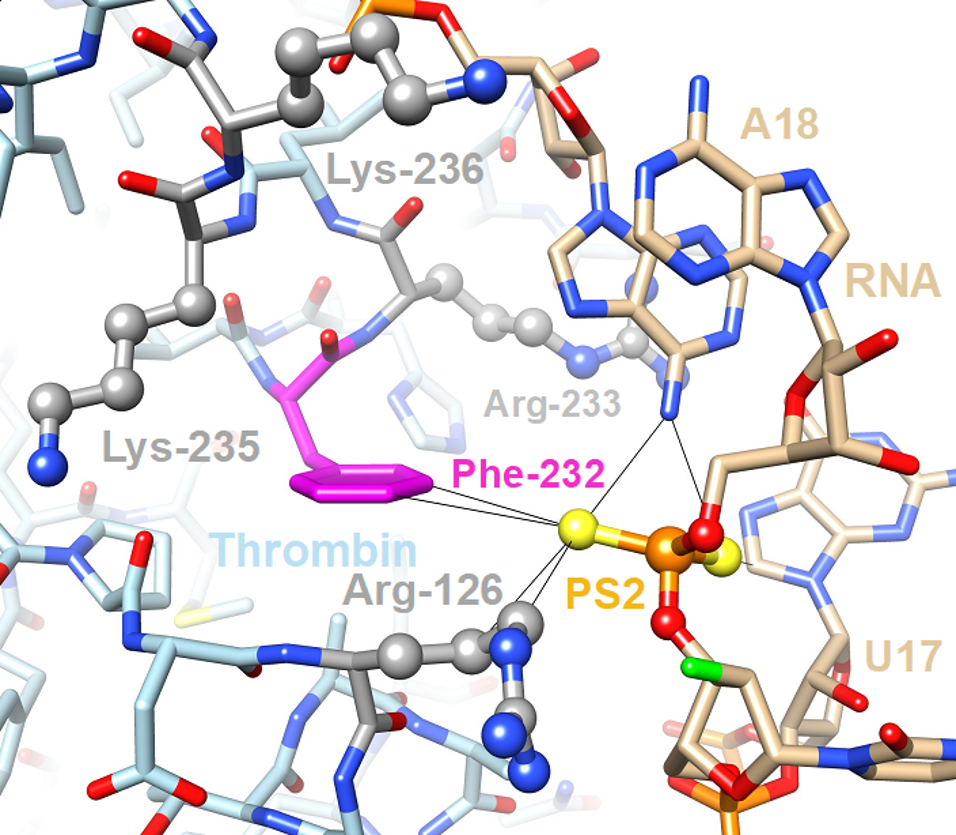
Close-up view of a key interaction between the PS2-modified antithrombin RNA aptamer and thrombin in the crystal structure of the complex (PDB ID 5DO4) [95]. An RNA-induced fit brings the PS2 moiety in close contact with the edge of Phe-232 (magenta carbon atoms) that forms a hydrophobic patch surrounded by four basic residues (side chains highlighted in ball-and-stick mode with carbon atoms colored in gray). These arginine and lysine residues generate an electric field that polarizes the thiophosphate moiety, thereby contributing to the 1000-fold tighter binding of the PS2-modified RNA to thrombin relative to the parent aptamer.

While the thermal stability of PS2-modified RNA duplexes slightly decreases compared to the unmodified duplex, there is an increase in nuclease stability, even relative to PS-modified duplexes [91]. Crystal structures of PS2-modified RNA duplexes were determined to be isomorphous to their native RNA counterpart, causing no perturbation in the ribose sugar conformation, nor the torsion angles of the backbone [92]. More interestingly, siRNA duplexes with PS2-modified sense strands showed an increase in binding affinity towards the Ago2 protein of the RISC complex [92–93]. The model based on the crystal structure of human Ago2 bound to an siRNA duplex demonstrated that PS2 moieties near the 3'-terminus of the sense strand lie in the vicinity of a hydrophobic patch that is surrounded by lysine and arginine residues [15]. The latter generate an electric field that could polarize sulfur atoms (the PS2 group still carries a negative charge), thereby enhancing the interaction of the PS2 moiety with the edge of phenylalanine as seen in the complex between PS2-modified anti-thrombin aptamer and thrombin [94] (Figure 3).

Commonly, internucleotide-modified oligonucleotides are coupled with 2'-substitutions in order to enhance or regain desirable therapeutic properties. For example, not only did introducing a 2'-*O*Me modification at the PS2 nucleotide sites of an siRNA duplex sense strand increase the thermal stability of the duplex to levels comparable to the unmodified variant, it also further improved the binding affinity to the Ago2 protein, hypothesized to be in part caused by a superior hydrophobic effect [92].

#### Glycol nucleic acid

**Scheme 3:**
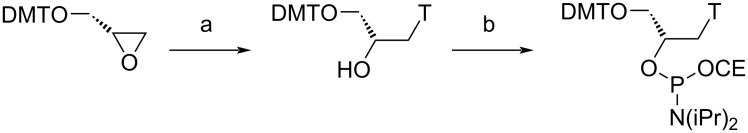
Synthesis of the (*S*)-GNA thymine phosphoramidite from (*S*)-glycidyl 4,4'-dimethoxytrityl ether. (a) Thymine, NaH, DMF; (b) ClP(NiPr_2_)(OCE), (iPr_2_)_2_NEt. T = thymine. Adapted from [99].

Glycol nucleic acid (GNA) with its chiral, acyclic three-carbon backbone linked by phosphate is the simplest phosphodiester-based nucleic acid analogue (Figure 1D). It contains one stereocenter allowing for the synthesis of either (*S*)-GNA or (*R*)-GNA where chirality is fixed by use of either (*R*) or (*S*) starting material, respectively. These simple nucleic acid building blocks were first synthesized in 1971 by Ueda et al. [96]. The group was able to synthesize adenine, cytosine, and uracil GNA analogues by reacting these bases with glycerol α-chlorohydrin or glycidol. The following year, the Seita group showed that thymine and guanine analogues could be prepared in a similar fashion [97]. Interestingly, both groups found that condensation of purine bases to yield GNA derivatives gave two dihydroxypropylated isomers: the N3 (I) and the N9 (II) dihydroxypropylated isomers. Using glycerol α-chlorohydrin, the ratio of I/II was 1:4 with II being the preferred isomer but when using glycidol, this ratio shifted to 3:1 in the favor of the desired isomer [96–97]. From there on, the use of glycidol for the preparation of GNA analogues became the gold standard. The first GNA polymers were obtained through condensation with *N*,*N*-dicyclohexylcarbodiimide (DCC) giving rise to homopolymeric tetramers of either G-GNA or T-GNA [97]. In 1996, Acevedo and Andrews were the first to demonstrate the synthesis of GNA nucleoside phosphoramidite derivatives as well as the ability of the phosphoramidite derivatives to withstand solid-phase conditions, inevitably laying the groundwork for GNA solid-phase synthesis [98]. Using the glycidol approach, Zhang et al. synthesized 18-mer oligonucleotides containing GNA-T monomers [99]. Starting from (*R*)-glycidol, the free hydroxy group is tritylated. The resulting product is then reacted with unprotected thymine which, in the presence of stoichiometric amounts of sodium hydride, results in the epoxide ring opening and the formation of the glycol backbone. The pre-amidite is then phosphitylated yielding the desired GNA-T amidite (Scheme 3). Recently, this simple acyclic nucleic acid backbone is of interest as a prospective evolutionary precursor of RNA [100]. Furthermore, GNA analogues with N2' → P3' phosphoramidate linkages have been studied as a potential alternative genetic system and they have been incorporated into siRNA duplexes to increase in vivo potency [54,100].

DNA oligomers containing GNA residues have been shown to form duplexes with DNA and RNA and to display self-pairing, whereby duplex formation was accompanied by hypochromicity [97,99]. In terms of stability, a single substitution from DNA to either (*S*)-GNA or (*R*)-GNA results in a decrease in *T*_m_ of 13 °C and 7 °C, respectively. As the number of substitutions is increased, the *T*_m_ decreases in a non-linear fashion. Replacement of all residues of a DNA strand by either (*S*)-GNA or (*R*)-GNA results in the complete loss of duplex formation, thereby confirming the detrimental effect of single and/or multiple GNA incorporations on duplex stability [101–102]. However, Zhang et al. demonstrated that an all-(*S*)-GNA can form a duplex with RNA [99]. It has been shown that a GNA/GNA duplex exceeds the thermal stability of DNA/DNA and RNA/RNA duplexes of the same sequence (increase in *T*_m_ of 18–25 °C) [99,101]. Moreover, (*S*)-GNA and (*R*)-GNA do not cross-pair either in a parallel or antiparallel fashion; thus GNA:GNA duplex formation is limited to homochiral pairing between either (*S*)-GNA or (*R*)-GNA strands [103]. With respect to nuclease stability, Nielson et al. showed that a 17mer oligonucleotide containing one T-GNA substitution has a nuclease half-life of 18–22 minutes in snake venom phosphodiesterase (SVPDE), thus exhibiting significantly higher stability compared to the parent strand [104]. Furthermore, Schlegel et al. showed that the position of the GNA substitution in a DNA/DNA duplex greatly influences its ability to resist 3'-exonucleases. Their work showed that a single or double (*S*)- or (*R*)-GNA substitution at the 3' end of a dT_20_ oligomer with a natural phosphodiester backbone greatly increases the oligonucleotide’s ability to resist SVPDE. Furthermore, when moving the single or dinucleotide substitution to the penultimate position, a marked decrease in nuclease stability was observed. However, when these modifications where moved to the terminal positions, an 8- or 5-fold increase in nuclease resistance was observed for the (*S*)- or (*R*)-isomer, respectively [54].

It is generally assumed that nucleic acid analogues require cyclic units in the backbone to generate the necessary conformational preorganization for duplex formation. This assumption does not hold true for GNA backbones where the destabilization caused by the shorter glycol moiety in DNA duplexes most likely stems from the structural incompatibility with the B-form deoxyribonucleotide-phosphate backbone. On the other hand, GNA–GNA duplexes form highly stable antiparallel duplexes that follow Watson–Crick base pairing rules [99]. GNA strands self-assemble into homochiral antiparallel right-handed ((*S*)-GNA) and left-handed ((*R*)-GNA) duplexes held together by Watson–Crick base pairs. Furthermore, these duplexes exhibit cross-strand base stacking consistent with A-form DNA and RNA duplexes [55].

**Figure 4:**
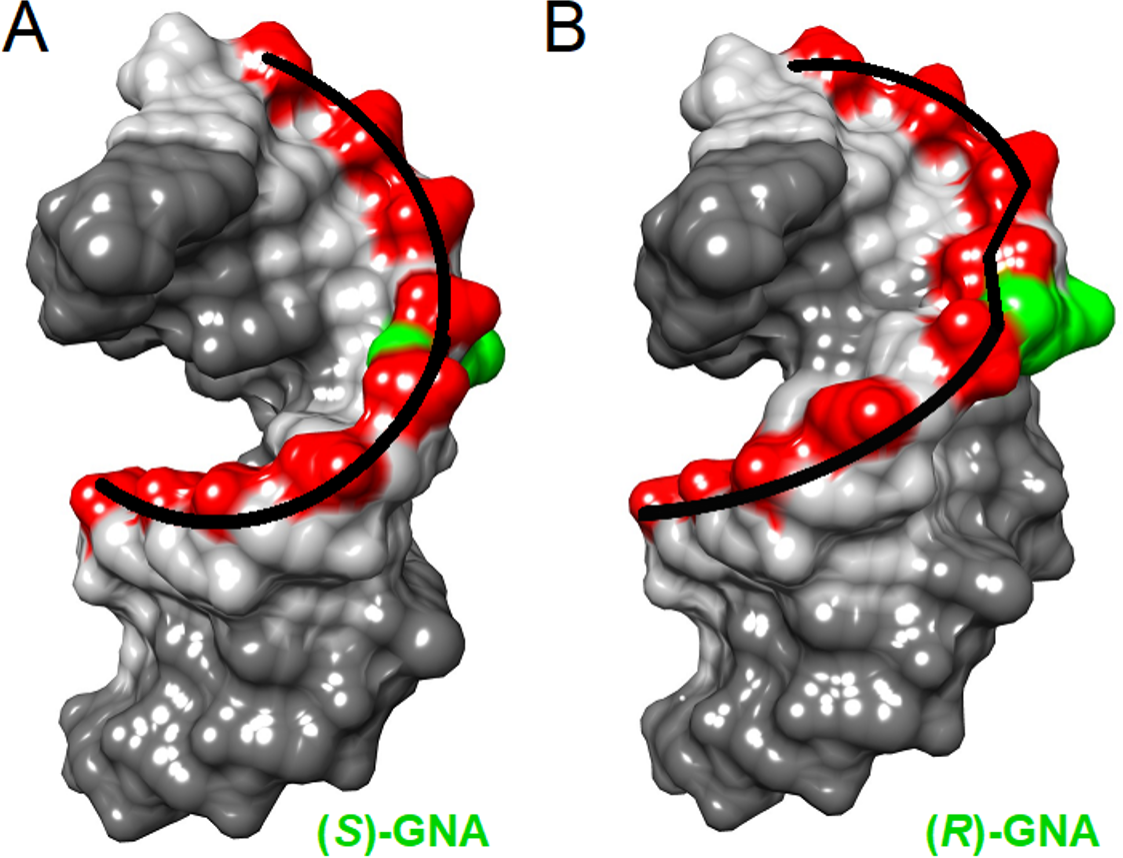
Surface models of the crystal structures of RNA dodecamers with single (A) (*S*)-GNA-T (PDB ID 5V1L) [54] and (B) (*R*)-GNA-T (PDB ID 5V1K) [54] nucleotides per strand. The presence of the (*R*)-GNA isomer introduces a kink in the backbone and causes local disruption of base stacking, in-line with a significantly reduced *T*_m_ relative to the (*S*)-GNA isomer.

Crystallographic studies have shown that (*S*)-GNA can form M-type helices (with metallo-base pairs) similar to A-form helices (with brominated base pairs). The M-type structure with 16 base pairs per turn and a helical pitch of 60 Å (ca. 3.8 Å helical rise) deviates significantly from the canonical A-form (11 base pairs/turn and ca. 2.6 Å rise) and B-form (10 base pairs/turn and ca. 3.4 Å rise) duplexes [54–55,105–107]. GNA duplexes possess only one large groove which corresponds to the canonical minor groove, the canonical major groove is a convex surface. Furthermore, the glycol backbone adopts two conformations alternating between *gauche* and *anti* conformations such that each base pair contains one nucleotide in the *gauche* conformation and one in the *anti* conformation. There is also a large backbone-base inclination (46° to −53°) which results in zipperlike interstrand and reduced intrastrand base stacking interactions [103]. The crystal structure of an RNA duplex containing (*R*)-GNA revealed that this modification disrupts both the phosphate backbone and hydrogen bonding of an adjacent base pair whereas (*S*)-GNA has a minimal influence on the structure of the duplex [54] (Figure 4). Moreover, incorporation of (*S*)-GNA residues in the seed region of the antisense strand of siRNA was observed to mitigate off target effects [54].

### Sugar and nucleobase modifications

#### 2'-O-Alkyl modifications

**Figure 5:**
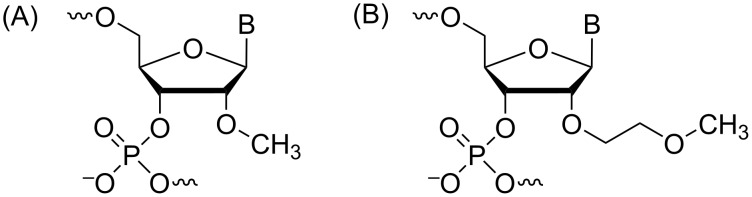
Structures of 2'-*O*-alkyl modifications. (A) 2'-*O*-methoxy RNA (2'-*O*Me RNA), (B) 2'-*O*-(2-methoxyethyl) RNA (2'-*O*-MOE RNA).

**Scheme 4:**
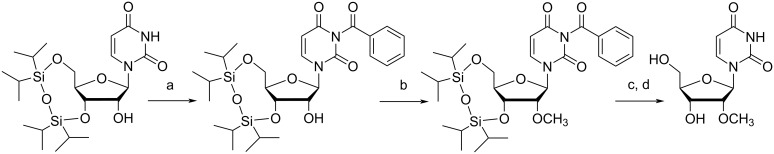
Synthesis of the 2'-*O*Me uridine from 3',5'-*O*-(tetraisopropyldisiloxane-1,3-diyl)uridine. (a) Benzoyl chloride, triethylamine; (b) CH_3_I, Ag_2_O; (c) dilute NH_4_OH; (d) 0.5 N HCl. Adapted from [108].

Historically, the 2'-*O*Me modification (Figure 5A) was the first of its class. The synthesis of each 2'-*O*Me ribonucleoside required specific considerations [108]. Starting from 3',5'-*O*-(tetraisopropyldisiloxane-1,3-diyl) (TIPDS) protected uridine, protection of N3 was needed in order to prevent methylation at this position (Scheme 4). The N3-benzoylated derivative could then be treated with methyl iodide in the presence of silver oxide in order to methylate the 2'-OH. A similar strategy was employed to synthesize 3',5'-*O*-TIPDS-*N*^4^-benzoyl-2'-*O*-methylcytidine. Next, 3',5'-*O*-TIPDS-*N*^6^-benzoyladenosine suffered from methylation at the nucleobase and thus, 6-chloro-9-β-ᴅ-ribofuranosylpurine was instead used as the starting material. Once TIPDS protected, the 2'-OH could, once again, be selectively methylated with methyl iodide and silver oxide. The protected adenine base was regenerated by treatment with ammonia followed by benzoylation. Once the methyl group was incorporated into these ribonucleosides, the TIPDS group was selectively removed by tetrabutylammonium fluoride (TBAF) or hydrochloric acid treatment, followed by 5'-tritylation. In the case of guanosine, this strategy for 2'-OH methylation was unsuccessful, owing again to undesired methylation at the nucleobase. Instead, the 5'-*O*-monomethoxytrityl derivative of *N*^2^-isobutyrylguanosine was treated with diazomethane in dimethylformamide in the presence of tin chloride, affording both 2'-*O*Me and 3'-*O*Me regioisomers. Fortunately, these isomers could be separated by silica gel column chromatography. Other synthetic approaches have since been developed [109–111], however, this pioneering work should be appreciated as nowadays, the 2'-*O*Me phosphoramidites of each protected ribonucleoside are all commercially available.

The study of 2'-*O*Me modified oligonucleotides was stimulated by the fact that they bind to RNA with higher affinity than unmodified RNA or DNA, as well as their improved nuclease resistance [112], promoting their usefulness in antisense therapies. Unfortunately, it was determined that uniformly 2'-*O*Me modified RNA:RNA duplexes were not substrates for RNAse H [113]. Structural insights of this modification were determined from the crystal structure of a duplex of self-complementary 10-mer DNA strands with a single internal 2'-*O*Me modified adenosine [114]. This duplex adopted an overall A-form, with the sugars in the C3'-*endo* orientation and the two, well solvated methoxy groups, pointing into the relatively wide minor groove of the duplex.

It was shown that as the number of carbons in the 2'-*O*-alkyl chain increased, so too did the destabilizing effect towards RNA binding affinity [115]. Thus, it was initially believed that even though nuclease resistance increased with chain length, this destabilizing effect would render 2'-*O*-alkyl-modified RNA a less potent therapeutic agent. In 1994, there was crystallographic evidence, however, that suggested the addition of a polarizable group in the longer 2'-*O*-alkyl chains that could hydrogen bond with nucleobases in the minor groove of the duplex would be well tolerated [114]. This supported the hypothesis that the 2'-*O*-[2-(methoxy)ethyl] (MOE) modification (Figure 5B) wouldn’t lead to significant destabilization of the duplex, prompting its development.

**Scheme 5:**
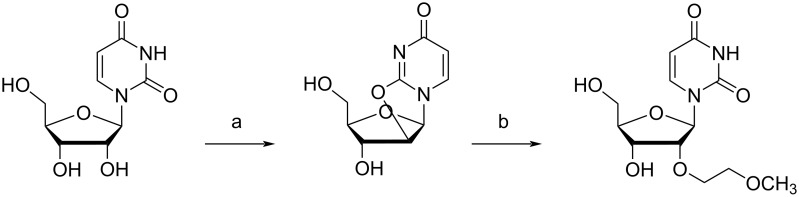
Synthesis of the 2'-*O-*MOE uridine from uridine. (a) (PhO)_2_CO, NaHCO_3_, DMA, 100 °C; (b) Al(OCH_2_CH_2_OCH_3_)_3_, reflux. Adapted from [117].

The synthesis of 2'-*O*-MOE-modified ribonucleosides was first described in 1995 [116]. Since then, two practical strategies have been developed for synthesizing 2'-*O*-MOE ribonucleosides. For pyrimidines, this involves treating 2,2'-anhydrouridine with aluminum 2-methoxyethoxide, which attacks and inserts at the 2'-position, opening the ring and producing the nucleoside with the correct stereochemistry (Scheme 5) [117]. Conveniently, this 2'-*O*-MOE uridine can be converted to the cytidine derivative by 4-nitrophenylation, 3',5'-trimethylsilylation and finally, treatment with aqueous ammonia. In contrast, the purine synthetic route first uses the bis-silylating agent [methylene bis(diisopropylsilyl)chloride] (MDPS) to protect both the 5' and 3'-hydroxy groups [118]. The protected nucleoside can then be treated with 2-methoxyethyl bromide in the presence of NaHMDS in order to selectively alkylate the 2'-OH, followed by TBAF treatment to remove the MDPS protecting group.

**Figure 6:**
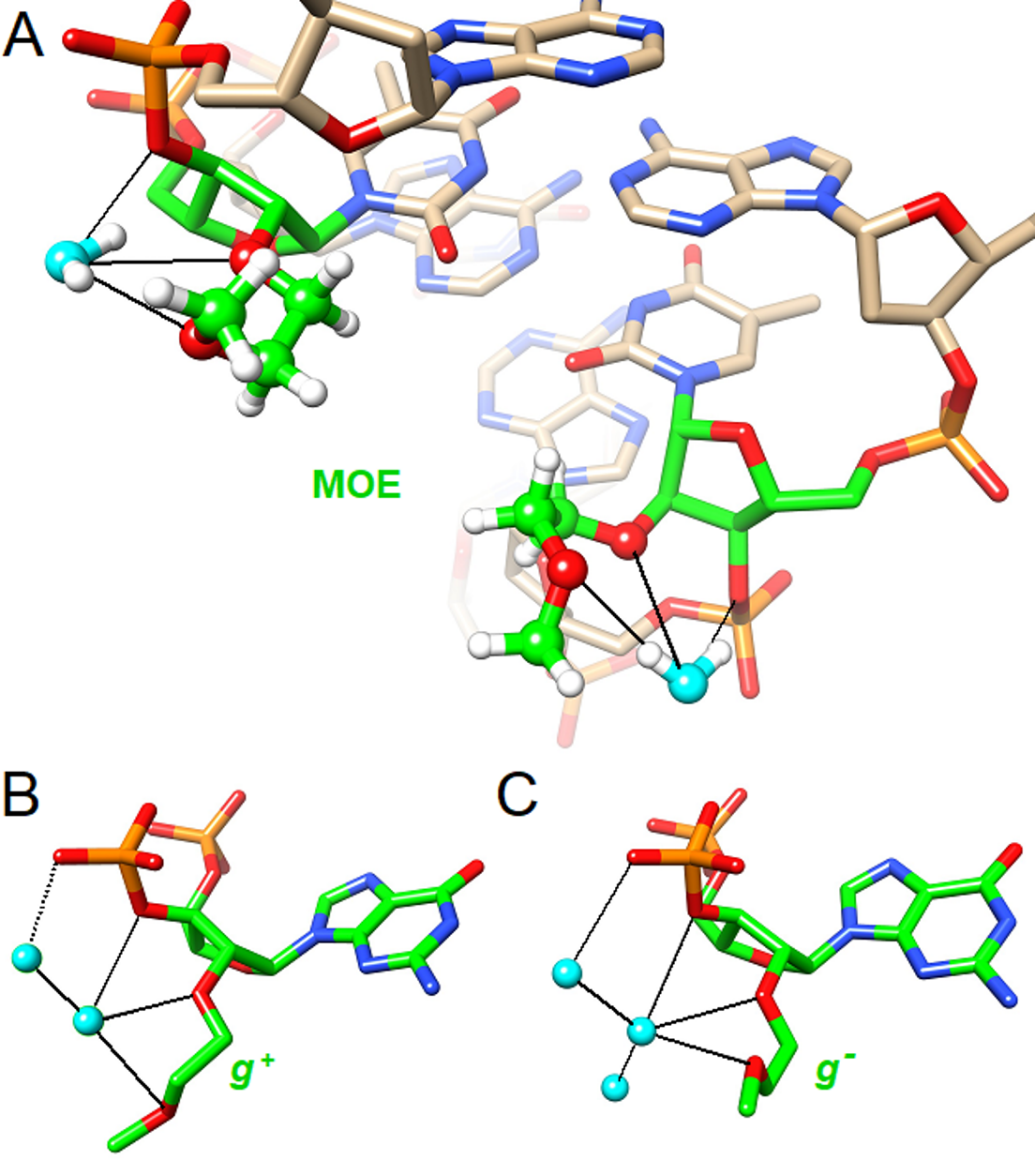
Structure of 2'-*O*-(2-methoxyethyl)-RNA (MOE-RNA). (A) View into the minor groove of an A-form DNA decamer with single MOE-T nucleotides per strand (PDB ID 411D, highlighted with green carbon atoms) [120]. Water molecules are trapped in a chelate-like manner between the O3', O2' and OC' (outer oxygen of the MOE substituent). (B) and (C) individual nucleotides from a crystal structure of an MOE-RNA dodecamer duplex (PDB ID 469D) [58]. Of the 24 MOE substituents, 22 adopt a gauche conformation, either *g*+ or *g*−, whereby both trap a water molecule that can be linked to the 3'-phosphate via a water bridge.

The 2'-*O*-MOE soon became the gold standard alkyl modification, owing to its improvement in therapeutically relevant properties. Compared to 2'-*O*Me RNA, the 2'-*O*-MOE RNA analogue has similar or even increased RNA binding affinity, as well as a tenfold increase in nuclease resistance [119]. Moreover, compared the PS-DNA, 2'-*O*-MOE RNA has an increased thermal stability of 2 °C per modification, with similar nuclease resistance [11,41]. Rationale for the improved properties of the 2'-*O*-MOE modification was gained through the analysis of the crystal structure of a uniformly modified self-complementary 12-mer RNA duplex [58]. The duplex was observed to be in the A-form, with the sugar residues being in a C3'-*endo* conformation. The MOE substituents were in the gauche orientation, being well accommodated in the minor groove and making a stabilizing interaction with a trapped water molecule and the adjacent phosphate (Figure 6). It’s this pre-organization of the MOE groups, making the duplex more rigid, which is hypothesized to cause the increase in RNA binding affinity. Furthermore, the increase in nuclease resistance is believed to be due to steric constraints from the MOE substituent and water molecule protecting the adjacent phosphate.

Many other 2'-*O*-alkyl modifications have been synthesized and studied extensively, and are summarized elsewhere [41,121]. Importantly, while 2'-*O*-alkyl-modified RNA cannot activate the RNAse H dependent degradation pathway, they can, however, act through a different therapeutic mechanism as steric blockers, inhibiting mRNA translation, RNA reverse transcription or RNA splicing [122–125].

#### Locked nucleic acids (LNA)/bridged nucleic acids (BNA)

**Figure 7:**
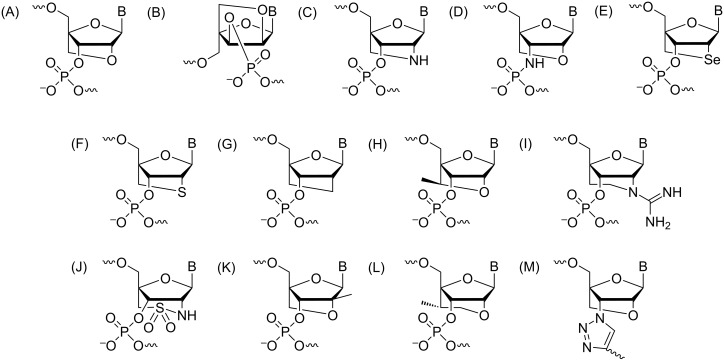
Structures of locked nucleic acids (LNA)/bridged nucleic acids (BNA) modifications. (A) LNA/BNA, (B) α-ʟ-LNA, (C) C2'-amino-LNA, (D) 3'-amino-2',4'-LNA, (E) seleno-LNA, (F) thio-LNA, (G) carba-LNA, (H) *S*-constrained ethyl (cEt) nucleic acid, (I) 2'-*N*-guanidino,4'-*C*-ethylene nucleic acid (GENA), (J) sulfonamide-bridged nucleic acid (suNA), (K) 2'-Me-LNA, (L) 6'-*S*-Me-2'-*O*,4'-*C*-ethylene-bridged nucleic acid (6'-*S*-Me-ENA), (M) triazole linked LNA.

**Scheme 6:**
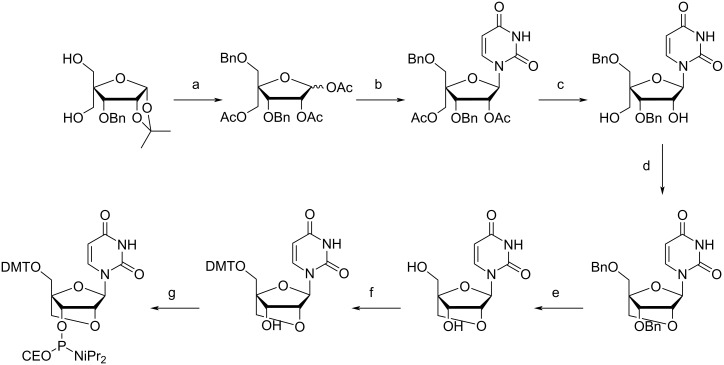
Synthesis of the uridine LNA phosphoramidite. (a) i) NaH, BnBr, DMF, ii) acetic anhydride, pyridine, iii) 80% AcOH, iv) acetic anhydride, pyridine; (b) uracil, *N*,*O*-bis(trimethylsilyl)acetamide, TMS-triflate, acetonitrile; (c) NaOCH_3_, methanol; (d) i) *p*-toluenesulphonyl chloride, pyridine, ii) NaH, DMF; (e) H_2_, Pd(OH)_2_/C, ethanol; (f) DMTCI, pyridine; (g) ClP(NiPr_2_)(OCE), (iPr_2_)_2_NEt, dichloromethane. Bn = benzyl, Ac = acetyl. Adapted from [128].

Locked nucleic acids are a class of modified nucleosides which traditionally involve the incorporation of a methylene bridge between C4' and O2' of the ribose sugar (Figure 7A). This incorporation, as first reported by both Wengel and Obika, locks the nucleoside in the C3'-*endo* (north) conformation which allows for enhanced binding affinities towards both DNA and RNA targets [126–127]. Both ^1^H NMR [127–129] and crystallographic studies [126] have been used to demonstrate the Northern puckering of the sugar and the *anti* orientation of the nucleobase. The key synthetic step in the synthesis of LNA involves the tosylation of a 4'-*C*-hydroxymethyl derivative, followed by a base-induced ring closure to afford the 2'-*O*,4'-*C*-linked bicyclic nucleoside derivative (Scheme 6) [127–128]. Incorporation of LNA into a variety of oligonucleotides with varying lengths and sequences has shown increased thermal stability when binding to either DNA or RNA complements with *T*_m_ increases of +1 to +8 and +2 to +10 °C, respectively [127–128,130–134]. The higher stabilization of RNA can be attributed to the preorganization of LNA nucleosides towards formation of A-form duplexes [128], whereas in DNA duplexes LNA residues steer the conformation of the neighboring DNA monomers into the C3'-*endo* conformation [135–136]. These modifications have also been shown to confer a higher level of nuclease resistance than isosequential DNA or phosphorothioate modifications [137–141]. In combination with the high selectivity for RNA sequences, this makes LNA-modified oligonucleotides well suited for use as antisense therapeutics. Recent publications have used LNA’s high affinity for RNA sequences in gapmer-designed antisense oligonucleotides for successful targeting of a key gene involved in TGFβ inhibition [142]. The inclusion of LNA nucleosides within a larger single-stranded DNA oligonucleotide has also allowed for subtle gene modifications to be implemented while evading mismatch repair (MMR) [143]. Furthermore, Ju et al. recently reported the use of LNA-based suppressors for the inhibition of viral miRNA through carbon dot-mediated delivery [144]. A diastereomer of LNA, α-ʟ-LNA (Figure 7B), also induces a higher affinity for both DNA and RNA complements in addition to providing a high stability against nucleases [145–146]. Unlike LNA, this diastereomer is a mimic of DNA instead of RNA and promotes a C2'-*endo* puckering of the sugar [147]. As a result, it has been shown to be better (fivefold) than other modified LNA analogues at knocking down target genes in vitro [145]. Also, these isomers have recently been shown to be useful in stabilizing streptavidin-binding aptamers [148], and in the use of antisense oligonucleotides for splice modulation through the induction of *Dmd* exon-23 skipping in mice in vitro [149].

Recently, a lot of attention has been paid to modifying the LNA scaffold to incorporate various heteroatoms, modify the bicyclic framework, and to change the location of the methylene bridge to tailor the properties of these nucleosides. The incorporation of nitrogen at C2' has been explored for further functionalization while retaining the LNA scaffold. Singh et al. were the first to report the synthesis of C2'-amino-LNAs (Figure 7C) in 1998 [150], with the synthetic route being optimized over time [151–152]. The stability of these derivatives is similar to those of LNA [150–152], with the added advantage of additional coupling reactions to fluorescent groups [151], or small molecules being possible either during solid-phase synthesis (SPS) [153–154] or post synthetically [155–156]. Gapmer oligonucleotides that incorporate 2'-amino-LNA show increased uptake in organs such as the heart, liver, and lungs in comparison to other LNA modifications [145]. Nitrogen can also be incorporated at the C3' position in the form of a 3'-amino-2',4'-LNA (Figure 7D) monomer which has been shown to stabilize oligonucleotides similarly to unmodified LNA with a nuclease resistance greater than PS-modified oligonucleotides [157]. Incorporation of selenium at C2' in a thymine-bearing LNA nucleoside (Figure 7E) has been demonstrated to have a hybridization ability and a nuclease resistance that are highly reversible in response to redox changes of the selenium atom [158]. Recent work has also looked at this modification in LNA nucleosides bearing an adenine base [159], but this nucleoside was found to be highly sensitive to heat, making its incorporation into oligonucleotides challenging. Thio-LNA (Figure 7F), which has sulfur incorporated at the C2' position, has similar binding properties as amino-LNA and β-ᴅ-LNA, but with varying biodistribution patterns and a higher cellular uptake in mice [145]. Work looking at carba-LNA, which lacks the O2' functionality, has shown the importance of the oxygen atoms in hybridizing to complementary RNA [160]. Unsubstituted carba-LNA (Figure 7G), which lacks a hydrophilic substituent at C2', leads to a decrease in heteroduplex stability [160]. This agrees with the observation in the crystal structure of an LNA-modified DNA duplex where the 2'-oxygen acts as an H-bond acceptor for water, potentially making a favorable contribution to the increased pairing affinity of LNA [161].

Constrained ethyl (cEt) nucleic acids (Figure 7H), which contain a [2.2.1] tricyclic core, have been developed and show improved potency when compared to second generation 2'-*O*-MOE antisense oligonucleotides [162–163]. The cEt also demonstrate an improved toxicity profile in comparison to standard LNA ASOs [162]. The arduous synthesis of the nucleoside analogues has been refined to minimize the number of needed stereochemical adjustments and overall steps [164]. ASOs containing these modified nucleosides have demonstrated promising antitumor activity for lymphoma and lung cancer [165].

Numerous other LNA analogues have been constructed including, but not limited to, 2'-*N*-guanidino,4'-*C*-ethylene (GENA) (Figure 7I) [166], sulfonamide-bridged (suNA) (Figure 7J) [167], 2'-Me LNAs (Figure 7K) [168–169], 6'-Me-2'-*O*,4'-*C*-ethylene-bridged (6'-Me-ENA) (Figure 7L) [170], and various triazole-linked LNA (Figure 7M) [171–172] that have all shown the ability to modulate LNA properties.

#### Arabinose and fluoroarabinose nucleic acids

**Scheme 7:**
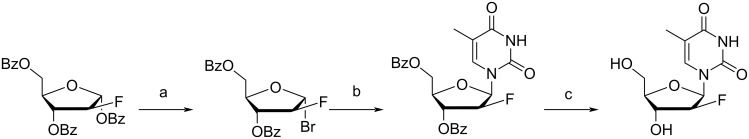
Synthesis of the 2'-fluoroarabinothymidine. (a) 30% HBr in acetic acid; (b) 2,4-bis-*O*-(trimethylsilyl)thymine, carbon tetrachloride; (c) NH_4_OH, methanol. Bz = benzoyl. Adapted from [177].

Arabino nucleic acids (ANA) are analogs of RNA where the hydroxy group at C2' is inverted (Figure 1G). In fluoroarabino nucleic acids (FANA) this C2' hydroxy group is replaced by fluorine. Arabino- and fluoroarabino nucleosides have demonstrated anticancer and antiviral activities (as reviewed in [173]). β-ᴅ-Arabinonucleosides of pyrimidines can be prepared from 2,2'-anydronucleosides [174] and purines from approaches which include condensation of the nucleobase with 2,3,5-tri-*O*-benzyl-ᴅ-arabinofuranosyl chloride [175]. The 2'-fluoro-β-ᴅ-arabinofuranose nucleosides can be prepared by coupling of the nucleobase with 3,5-di-*O*-benzoyl-2-deoxy-2-fluoro-α-ᴅ-arabinofuranosyl bromide (Scheme 7) [176–180]. Both β-ᴅ-arabino and 2'-fluoro-β-ᴅ-arabinofuranose nucleosides can be converted to phosphoramidite derivatives for incorporation into oligonucleotides for solid-phase synthesis [178,181–184].

Hybridization studies of uniformly modified ANA of mixed nucleobase composition to complementary RNA revealed reduced thermal stability relative to the corresponding DNA/RNA duplex by approximately 1.5 °C per modification [182–183]. A significant reduction in stability of the duplex was observed in the binding of ANA to complementary DNA relative to the DNA duplex [182–183]. In contrast, FANA of mixed nucleobase composition displayed improved binding with both complementary DNA and RNA, relative to DNA/DNA and DNA/RNA duplexes by approximately 1 °C and 0.5 °C per modification, respectively [178]. The 2'-stereoisomer of FANA, FRNA also demonstrates improved binding to RNA, relative to DNA [185]. Circular dichroism spectra of FANA/RNA and ANA/RNA duplexes show similarity to that of DNA/RNA [178,183]. Both ANA and FANA demonstrate good stability to nucleases [183,186]. Hybrid duplexes of ANA and FANA with complementary RNA were substrates of RNase H, with greater cleavage of the RNA strand observed for the latter, demonstrating the gene silencing potential of these analogs [183,186]. Uniformly modified phosphorothioate (PS) FANA forms a duplex with RNA with a higher *T*_m_ relative to the PS-DNA/RNA duplex, however, RNase H-mediated cleavage of RNA was diminished for the duplex formed with PS-FANA relative to PS-DNA [187]. Improved cleavage by RNase H was observed with chimeric PS-FANA/DNA [187]. PS-FANA/DNA chimera with either flanked or alternating segments of FANA residues, as demonstrated by knockdown of *c-MYB* mRNA with a persistent silencing effect [188].

**Figure 8:**
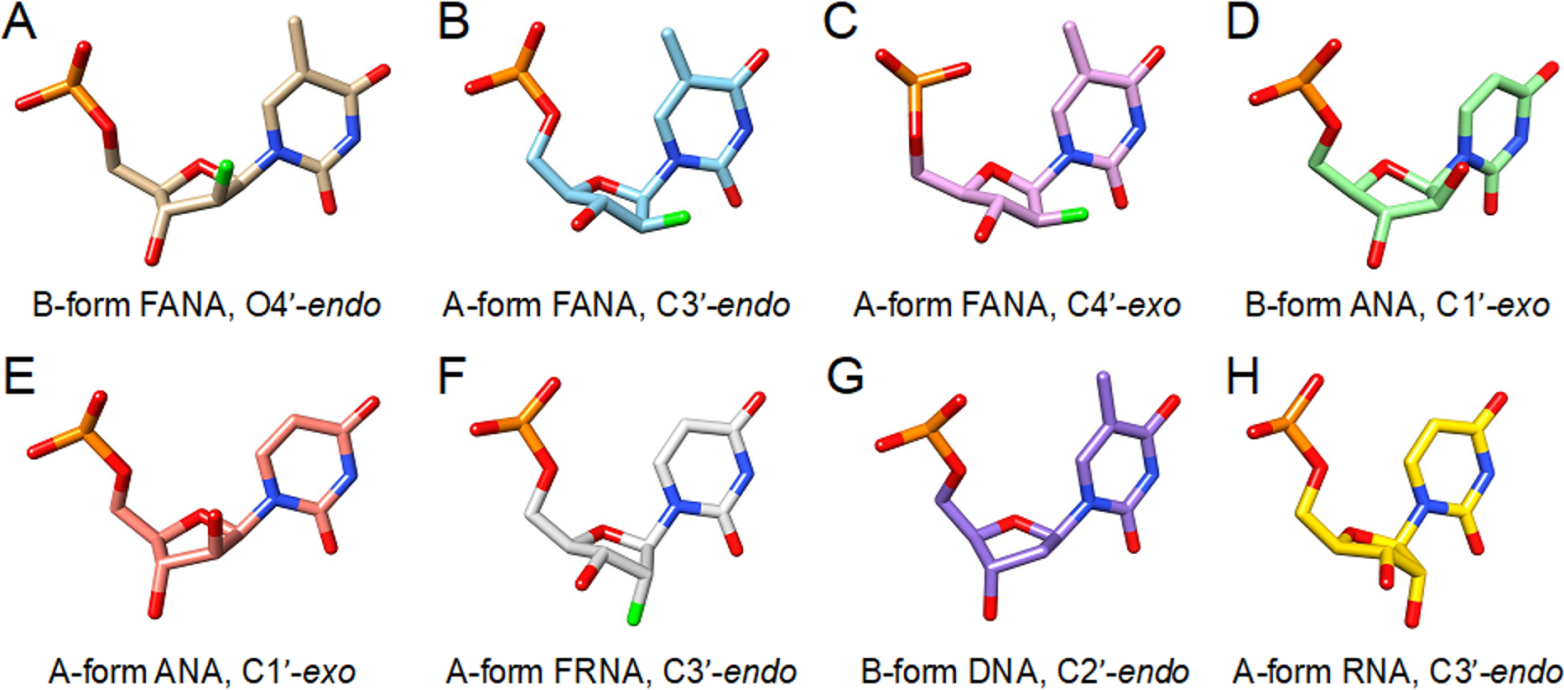
Sugar puckers of arabinose (ANA) and arabinofluoro (FANA) nucleic acids compared with the puckers of the fluoro-ribonucleic acid analog (FRNA) as well as DNA and RNA. (A) FANA-T in B-form DNA (PDB ID 388D) [189]. (B) FANA-T in A-form DNA (PDB ID 2FIL, duplex 1) [190]. (C) FANA-T in A-form DNA (PDB 2FIL, duplex 2) [190]. (D) ANA-U in B-form DNA (PDB ID 2FII) [190]. (E) ANA-U in A-form DNA (PDB ID 2FIJ) [190]. (F) FRNA-U in A-form RNA (PDB ID 3P4A) [62]. (G) B-form DNA (PDB ID 388D) [189]. (H) A-form RNA (PDB ID 5DEK) [199].

A 1.55 Å crystal structure of a Dickerson–Drew dodecamer containing fluoroarabinothymine revealed that these modified nucleotides adopt an O4'*-endo* (east) conformation that is readily accommodated in a B-form duplex [189] (Figure 8A). Fluoroarabinothymine in an A-form DNA duplex had a northern conformation (Figure 8B,C) whereas arabinouridine in either an A- or B-form environment had a south-eastern conformation (Figure 8D,E), suggesting greater flexibility for FANA versus ANA [190]. NMR structures of hairpin duplexes consisting of RNA and either FANA or ANA stems suggested that both modifications adopt an O4'-*endo* sugar pucker [191–192]. The O4'-*endo* sugar conformation has been reported for the DNA strand in DNA/RNA hybrid duplexes, the natural substrate of RNase H [193–194]. Structures of duplexes containing FANA and FRNA (Figure 8F) have revealed that thermal stabilization may be attributed to nonconventional hydrogen bonds in the backbone [195–197]. Gene silencing by RNAi has also been explored with siRNA containing FANA residues [198]. These studies have shown that FANA is accommodated in the sense strand and 5'-end and 3'-termini of the antisense strand of the siRNA [198].

#### C4'-Modified nucleic acids

**Figure 9:**
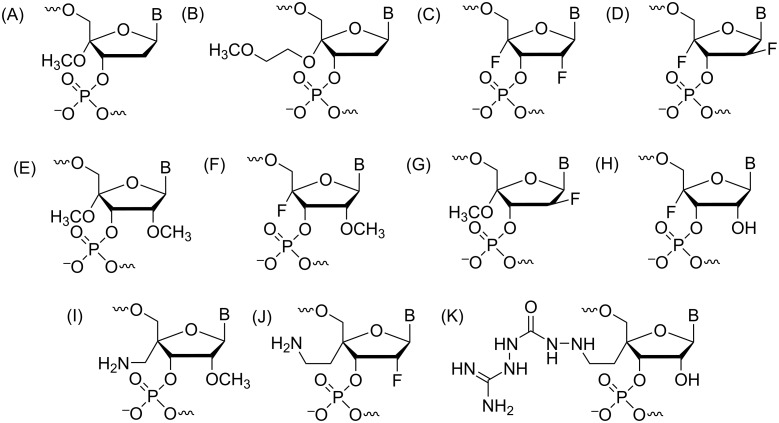
Structures of C4'-modified nucleic acids. (A) 4'-methoxy, (B) 4'-(2-methoxyethoxy), (C) 2',4'-difluoro (2',4'-diF) RNA, (D) 2',4'-difluoro (2',4'-diF) ANA, (E) 2',4'-dimethoxy RNA, (F) 2'-methoxy,4'-fluoro RNA, (G) 2'-fluoro,4'-methoxy ANA, (H) 4'-fluoro RNA, (I) 4'-*C*-aminoalkyl-2'-*O*-methyl, (J) 4'-*C*-aminoalkyl-2'-fluoro, (K) 4'-*C*-guanidinocarbohydrazidomethyl.

Modifications at the C4' sugar position (Figure 1H) have long been desirable as a means of modulating the properties of nucleic acids without interfering with Watson–Crick pairing. Incorporations at C4' are close in proximity to both the 3' and 5'-neighboring phosphate groups, allowing for a tailoring of the nuclease resistance [200]. In 2011, Rosenberg demonstrated the favorable binding properties of an oligothymidylate modified with 4'-methoxy or 4'-(2-methoxyethoxy) functionalities (Figure 9A,B) [201]. These modified nucleic acids were found to have superior hybridization behaviors towards both complementary DNA (see Figure 8G for pucker) and RNA (see Figure 8H for pucker) with sugar puckers in the northern (C3'-*endo*) and southern (C2'-*endo*) configurations for the respective alpha and beta isomers [201]. In 2015, this work was extended to incorporate these modifications into oligonucleotides containing all four bases [202]. *N*-Iodosuccinimide promoted the alkoxylation of the 4'–5'-enol acetates yielded the corresponding 5'-acetoxy-5'-iodo-4'-methoxy intermediates [202]. These intermediates were hydrolyzed with a mixture of triethylammonium bicarbonate (TEAB) and *N*,*N*-dimethylformamide (DMF) followed by a sodium borohydride reduction to give the 4'-alkoxy products [202]. The 4'-methoxy-2'-deoxynucleosides exhibited high resistance towards depurination under acidic conditions [202]. In contrast, nucleosides that are modified with 4'-fluoro modifications have more labile glycosidic linkages under similar conditions [203–204]. Rosenberg attributed this contrast to the electronegativity differences between the groups and the effect this would have on the stabilization of the resulting oxocarbenium ion [202]. Oligomers modified with the 4'-methoxy modification hybridized better to complementary RNA, rather than DNA, due to the N-type conformation of the sugar pucker, as confirmed by NMR [202]. These same oligomers exhibited half-lives of approximately 40 minutes in the presence of phosphodiesterase I [202]. In contrast, the natural DNA sequence had a half-life of 1 min [202].

The incorporation of fluorine at the C4' position has long constituted a challenge owing to the instability of the glycosidic bond in the resulting nucleosides. This modification is desirable due to its involvement in the mode of action of the natural antibiotic nucleocidin [203,205]. Damha reasoned that the incorporation of fluorine at both C2' and C4' could lead to a stable nucleoside due to the glycosidic bond stabilization brought about by 2'-fluorination [206] which turned out to be correct after successful isolation of both 2',4'-diF-rU and 2',4'-diF-rC nucleosides (Figure 9C) [206]. Through NMR, these nucleosides were found to be essentially locked in the northern (C3'-*endo*) sugar pucker, albeit without the need for the bicyclic structures typical for locked nucleic acids [206]. The 2',4'-diF-rU nucleoside was introduced into an RNA by way of an HCV polymerase and extended to give a full-length oligonucleotide product, whereas 2',4'-diF-rUTP inhibited RNA synthesis at the early stages of dinucleotide-primed reactions [206]. Standard solid-phase synthesis allowed for the incorporation of this modified nucleoside into both RNA and DNA oligonucleotides. The impact on stability was found to be minimal in the case of RNA/RNA duplexes; mildly destabilizing with RNA/DNA hybrid duplexes; and highly destabilizing when incorporated into the DNA strand of DNA/RNA or DNA/DNA duplexes [207]. Damha attributed this destabilization to structural distortions caused by A/B junctions within the helical structures [207].

2',4'-diF-modified siRNA sequences were capable of triggering RNAi with high efficiency, and the incorporation of multiple residues in the guide (antisense) strand yielded more potent siRNAs than those containing LNA or FANA modifications [207]. 2',4'-diF-ANA (Figure 9D) also adopted the northern (C3'-*endo*) sugar pucker despite the 2'-βF, which generally leads to the adoption of a southern or eastern pucker [208]. This monomer was found to have minimal effects on the thermal stability of nucleic acid duplexes. However, when incorporated into a DNA/RNA hybrid duplex it was shown to decrease the rate of both human and HIV reverse transcriptase-associated RNase H-mediated cleavage [208]. In 2018, the work was expanded to include 2',4'-diOMe-rU, 2'-OMe,4'-F-rU, and 2'-F,4'-OMe-araU nucleosides (Figure 9E,F,G) [209]. This work reinforced the notion that both 4'-OMe and 4'-F modifications steer the sugar pucker towards a C3'-*endo* (north) conformation [209], even in the presence of C2' groups that would favor a different puckering of the ribose sugar. The 4'-modifications provided either a small stabilizing or destabilizing effect depending on the type of underlying duplex, and these 4'-substituents were able to modulate the binding affinities for the parent 2'-modifed oligonucleotides [209]. siRNA containing inserts of the C4’ α-epimer of 2'-F,4'-OMe-rU, in either the sense or antisense strands, triggered gene silencing with efficiencies comparable to that of 2'-F-rU [210].

**Scheme 8:**
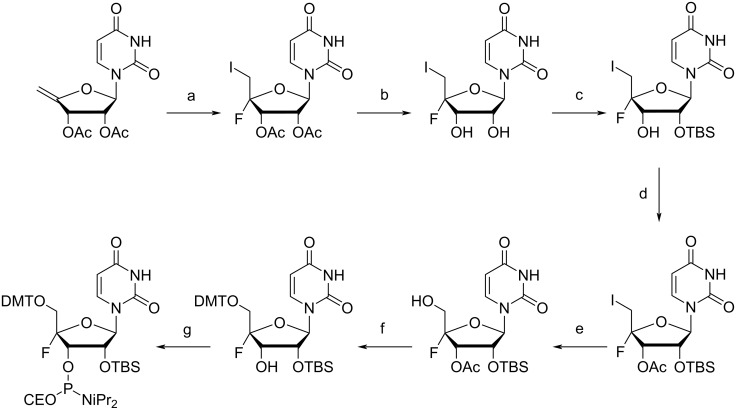
Synthesis of the 4'-F-rU phosphoramidite. (a) AgF, I_2_, dichloromethane, tetrahydrofuran; (b) NH_3_, methanol; (c) TBS-Cl, AgNO_3_, pyridine, tetrahydrofuran; (d) acetic anhydride, dimethylaminopyridine, pyridine; (e) tetrabutylammonium hydroxide, trifluoroacetic acid, *m*-chloroperoxybenzoic acid; (f) (i) NH_3_, methanol (ii) DMTCI, pyridine; (g) ClP(NiPr_2_)(OCE), 1-methylimidazole, (iPr_2_)_2_NEt, dichloromethane. TBS = *tert*-butyldimethylsilyl. Adapted from [211].

Recently, Zhou provided the first synthesis of a 4'-F-rU (Figure 9H) phosphoramidite which was stable enough to then be incorporated into longer oligonucleotides through standard solid-phase synthesis (Scheme 8) [211]. They found that the modified 4'-F-rU ribonucleotide had a high resemblance to the unmodified uridine, allowing it to be used as a probe for RNA structure determination through ^19^F NMR [211]. This modification led to RNA which was stable and predominantly in the C3'-*endo* (north) conformation [211], similar to the 2',4'-diF-RNA previously reported by Damha [208]. Zhou reasoned that because 3'-*O*-β-glucosylated nucleocidin, an intermediate in the biosynthetic pathway of nucleocidin, was stable, they may be able to successfully achieve the synthesis of the 4'-F-rU phosphoramidite through a selective protection of the hydroxy groups in stages [211]. Starting with a prepared 5'-iodo-4'-fluorouridine analogue that had been used in previous attempts of this synthesis, they removed the acetyl protecting groups at C3' and C2' with NH_3_/MeOH to give 5'-iodo-4'-fluorouridine [211]. Selective protection of the 2'-OH with TBDMS-Cl followed by protection of the 3'-OH with an acetyl group gave the fully protected intermediate [211]. Treatment of this intermediate with m-CPBA in the presence of a phase-transfer catalyst in acidic medium gave the resulting 5'-OH compound [211]. The authors reported no transfer of the 2'-TBDMS group onto the 5'-OH, however, following removal of the 3'-*O*-acetyl group with NH_3_/MeOH, some TBDMS transfer to the C3' position is seen [211]. 5'-DMT protection then led to the pre-amidite [211]. ^19^F NMR results show that not only does this modification allow for discernment between ssRNA and dsRNA, but it also allows for the identification of mismatches and the binding of RNA-processing proteins with chemical shift dispersions as large as 4 ppm, suggesting that this modification has a wide use for the determination of a variety of RNA structures through NMR spectroscopy [211].

In contrast, the incorporation of 4'-*C*-aminoalkyl-2'-*O*-methyl (Figure 9I) nucleosides leads to a slight destabilization of helical structures due to the adoption of a C2'-*endo* (south) conformation [212–213]. When fluorine is incorporated at C2' instead of 2'-*O*Me (Figure 9J), these 4'-*C*-aminoalkyl nucleosides are found to stabilize both dsRNA and siRNA to a larger extent [214]. The incorporation of 8 nucleosides into an siRNA passenger strand showed RNAi activity identical to the unmodified siRNA, with 50% of the siRNA strands remaining intact after 48 h in 20% BSA [214]. Recent work on the synthesis of novel 4'-*C*-guanidinocarbohydrazidomethyl-5-methyluridine (GMU) (Figure 9K) has shown that functionalizing the C4' position with guanidinium leads to siRNAs with increased thermal stability (1–3 °C/mod) and improved stability in human serum [215]. These guanidinium-modified siRNAs also lead to sustained gene silencing with only picomolar concentrations after 96 h of transfection [215]. Their qPCR experiments show that the cause of this sustained gene silencing activity is due to enhanced guide strand recruitment within the RISC complex [215].

#### 3'-Fluorohexitol nucleic acids (FHNA)

Herdewijn was the first to describe the synthesis as well as the biophysical, structural, and biological characterization of hexitol nucleic acids (HNA), mannitol nucleic acids (MNA), and altritol nucleic acids (AtNA) [216–220]. These carbohydrate-modified nucleosides incorporate a six-membered pyranose ring in place of the furanose ring found in unmodified DNA and RNA, with the nucleobase positioned at the C2' position in an axial orientation mimicking the C3'-*endo* (north) sugar puckering of furanose nucleosides [221]. MNA and AtNA possess an additional hydroxy group at the C3' position in the *R* and *S* configurations, respectively [219–220]. HNA was found to bind to complementary RNA in an antiparallel, sequence-dependent fashion, leading to the stabilization of HNA/RNA duplexes [218]. HNA also stabilizes HNA/DNA duplexes but to a smaller degree due to differences in minor groove solvation [222]. mRNA translation experiments have shown that HNA can function as a steric blocking agent of Ha-ras in cell-free experiments [223]. AtNA/RNA displays higher thermal stability when compared to HNA/RNA and natural nucleic acid controls [220]. In contrast, the introduction of MNA leads to duplex destabilization due to unfavorable steric clashes and limited nucleoside preorganization [219].

**Scheme 9:**
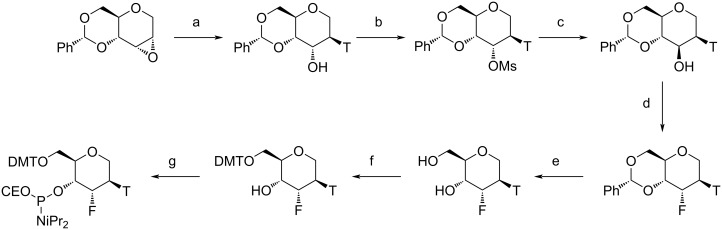
Synthesis of the thymine FHNA phosphoramidite. (a) thymine, 1,8-diazabicyclo[5.4.0]undec-7-ene, acetonitrile; (b) methanesulfonyl chloride, pyridine; (c) aq NaOH, 1,4-dioxane; (d) nonafluorobutanesulfonyl fluoride, 1,8-diazabicyclo[5.4.0]undec-7-ene, tetrahydrofuran; (e) H_2_, Pd(OH)_2_/C, methanol; (f) DMTCI, pyridine; (g) P(NiPr_2_)_2_(OCE), 1*H*-tetrazole, NMI, DMF. Ms = methanesulfonyl, Ph = phenyl, T = thymine. Adapted from [221].

**Scheme 10:**
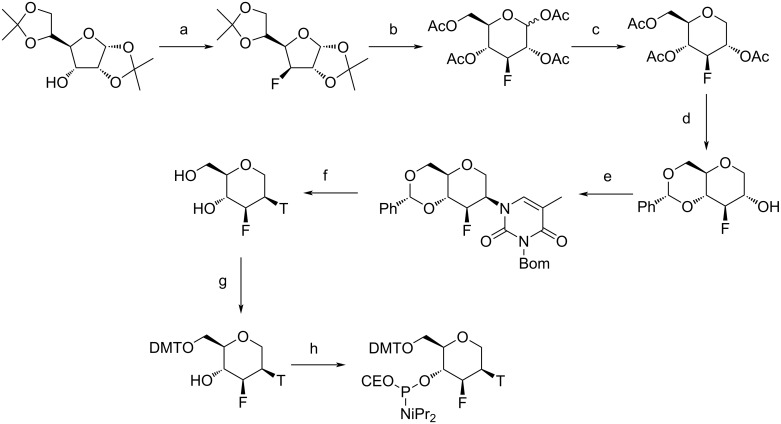
Synthesis of the thymine Ara-FHNA phosphoramidite. (a) i) trifluoromethanesulfonic anhydride, pyridine, ii) CsF, *tert*-butanol; (b) i) Amberlite IR-120-H, 1,4-dioxane, water, ii) acetic anhydride, pyridine; (c) i) 33% HBr in acetic acid, dichloromethane, ii) tributyltin hydride, 2,2′-azobis(2-methylpropionitrile), toluene; (d) i) K_2_CO_3_, methanol, ii) benzaldehyde dimethyl acetal, *p*-toluenesulfonic acid, DMF; (e) i) trifluoromethanesulfonic anhydride, dichloromethane, pyridine, ii) N3-benzyloxymethylthymine, 1,8-diazabicyclo[5.4.0]undec-7-ene, dimethyl sulfoxide; (f) H_2_, Pd(OH)_2_/C, methanol; (g) DMTCI, pyridine; (h) P(NiPr_2_)_2_(OCE), 1*H*-tetrazole, NMI, DMF. Bom = benzyloxymethyl. Adapted from [221].

In 2011, a work detailing the first synthesis of both isomers of 3'-fluoro-modified hexitol nucleic acid (FHNA and Ara-FHNA) (Figure 1I) was published (Scheme 9 and Scheme 10) [221]. The incorporation of fluorine has long been used in siRNA [224], miRNA [225], and for ^19^F NMR structural studies of nucleic acids [211]. It was proposed that the incorporation of fluorine at the C3' position of HNA could further expand its use as a potential antisense therapeutic [221].

**Figure 10:**
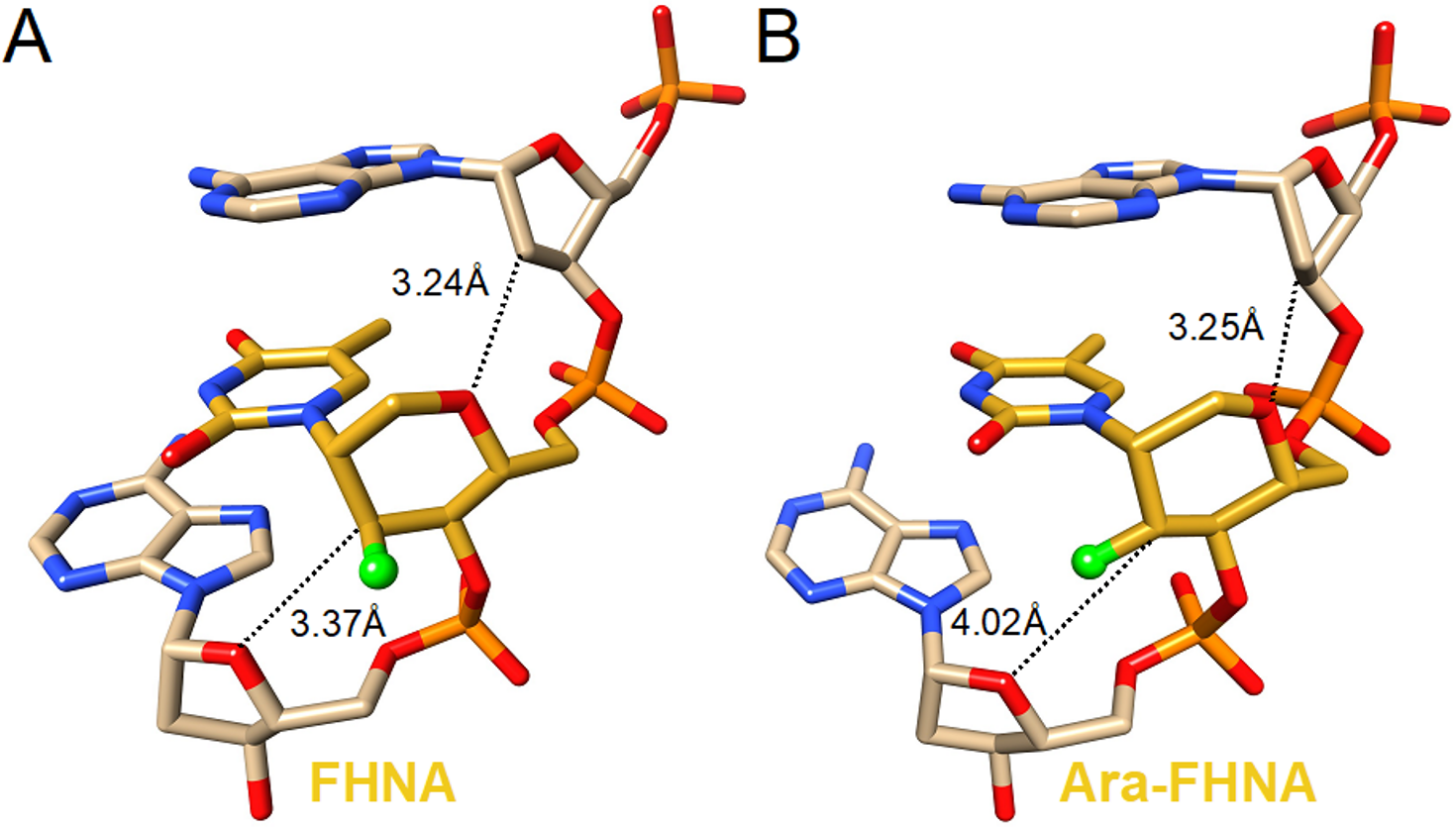
Crystal structures of (A) FHNA and (B) Ara-FHNA in modified A-form DNA decamers (PDB IDs 3Q61 and 3SD8, respectively) [221]. Unlike the trans-diaxial orientation of the fluorine in FHNA, the equatorial orientation of fluorine in Ara-FHNA pushes away the 3'-adjacent nucleotide (dashed lines) and causes local unstacking of bases.

The published data show that incorporation of a 3'-fluorine atom in the trans-diaxial orientation relative to the base in FHNA (Figure 10A) leads to stabilization of the resulting nucleic acid duplex, whereas the incorporation of ara-FHNA leads to sequence-dependent destabilization of the duplex [221]. The FHNA modification is better at discerning G–T mismatches than DNA or LNA, and both FHNA and Ara-FHNA were more stable against exonuclease digestion in comparison to LNA and MOE-modified oligonucleotides [221]. X-ray crystallographic studies showed that the equatorial 3'-fluorine of Ara-FHNA-T in the A-form DNA decamer pushes away O4' from the 3'-adjacent 2'-deoxy-A within the minor groove of the duplex [221] (Figure 10B). To avoid a clash between the Ara-FHNA hexose and the 3'-adjacent deoxyribose, the duplex undergoes a slight conformational change that results in partial unstacking of the thymine and adenine bases [221], explaining the lower RNA affinity of Ara-FHNA compared to FHNA. Further experiments in vivo also demonstrated the effectiveness of FHNA-modified siRNA in the downregulation of mouse phosphatase and tensin homologue (PTEN) without inducing hepatotoxicity [221]. Recent work has also shown that FHNA modifications improve the potency of GalNAc-conjugated gapmer ASOs [226].

Methylation at the C6' position further influences the RNA affinity of nucleic acids containing these modifications. *R*-6'-Me-FHNA is highly destabilizing, whereas *S*-6'-Me-FHNA leads to duplex stabilization [227]. This trend is identical to the C5' methylation of LNA [228]. The 1.24 Å crystal structures of A-form decamer duplexes containing these C6'-methylations show a small 1–5 intranucleoside contact between the C6' methyl group and the O4' in *R*-6'-Me-FHNA [227]. Additionally, *R*-6'-Me-FHNA perturbs the structure of water surrounding the O2P atoms which will further reduce the pairing affinity of the *R* isomer [227].

Herdewijn recently published the synthesis of 4'-aminotritylhexitol nucleosides for the eventual synthesis of N4' → P6' phosphoramidates of aminohexitol nucleic acids (AHNA) [229], as well as the synthesis of 3'-fluoro-4'-aminohexitol nucleosides which contain both the 3'-fluoro functionality and the N4' → P6' phosphoramidate linkage [230].

#### Ribo-difluorotoluyl

2'-Deoxydifluorotoluyl (dF) nucleoside derivatives (Figure 1J) were first synthesized by Schweitzer and Kool in 1994 in order to study the importance of H-bonding and base stacking in DNA. Specifically, they focused on the 2,4-difluorotoluene moiety as an isostere of the natural thymine base, albeit without the ability to form H-bonds [231]. A few years later, in 1997, Moran et al. showed that dF was a good template for enzymatic DNA synthesis, permitting production of the complementary DNA strand and hence suggesting that shape complementarity may be more important than H-bonding for fidelity and efficiency of DNA polymerases [232–233]. Recently, the rF nucleoside analogue has been investigated for its ability to efficiently silence gene expression when incorporated into short interfering RNA (siRNA) duplexes and to further investigate the fidelity of various RNA polymerases [234–236]. siRNA guide strands modified at the 5' end with rF showed similar silencing to the unmodified control. Furthermore, internal rF modifications showed lower affinity for their target but exhibited higher nuclease resistance [235,237]. Moreover, the rF/A pair lowers the *T*_m_ of the siRNA duplex but is less destabilizing than a mismatch (A/A, C/A and G/A) [235]. Several crystal structures of oligonucleotides containing the dF or rF nucleoside analogue alone and oligos with dF bound to DNA polymerases have been determined [235,237–240]. The 1.6 Å resolution structure of the Dickerson–Drew dodecamer (DDD) with dF replacing T8 (i.e., dCGCGAATFCGCG), solved with crystals of the duplex grown in the presence of *Bacillus halodurans* RNase H (which was bound to the duplex but did not exert an influence on its structure), revealed distances of 3.09 and 3.12 Å for the F^4^(dF)···N^6^(A) atoms of the two dF:A pairs similar to the O^4^(T)···N^6^(A) distance (2.96 and 3.11 Å) observed for the native DDD [240]. The 1.6 Å crystal structure of a duplex containing the rF analog ([rCGCFAAUUAGCG]_2_) revealed a F^4^(rF)···N^6^(A) distance of approximately 4 Å between the rF:A pairs [235].

## Conclusion

Chemically modified oligonucleotides have come of age as a class of therapeutic agents for a number of diseases. Taking inspiration from the structure, properties and biological roles of nucleic acids, scientists have employed chemistry to prepare a diverse collection of modifications to the architecture of this molecule imbuing desirable characteristics for applications as a therapeutic agent. In addition, many nucleic acid analogs have been explored for additional studies including investigation of artificial genetic systems, catalysts, and sensors. Amongst the oligonucleotide-based therapeutics that have been approved as drugs, the dominating modifications are the phosphorothioate backbone and at the C2'-position (of ribose) including 2'-*O*Me, 2'-F, and 2'-*O-*MOE. Moreover, combinations of these modifications in an oligonucleotide leads to a synergistic effect enhancing their therapeutic properties. Such combinations of nucleotide and backbone modifications with numerous analogs that have been developed will continue as an exciting direction for the next generation of oligonucleotide-based therapeutics. Rational design of future modifications with improved properties may be gleaned from insights from structural techniques. For example, stability, gene silencing and structural studies of chemically modified oligonucleotides containing fluorine at the sugar and nucleobase have provided insights into the role of noncovalent interactions on the properties of these molecules. The partnership between organic synthesis, biophysical chemistry, biochemistry and structural biology continues to guide the design and drive the achievements for oligonucleotide-based therapeutics.

## References

[1] Sood A J, Viner C, Hoffman M M (2019). J Cheminf.

[2] Boccaletto P, Machnicka M A, Purta E, Piątkowski P, Bagiński B, Wirecki T K, de Crécy-Lagard V, Ross R, Limbach P A, Kotter A (2018). Nucleic Acids Res.

[3] Wagner M, Steinbacher J, Kraus T F J, Michalakis S, Hackner B, Pfaffeneder T, Perera A, Müller M, Giese A, Kretzschmar H A (2015). Angew Chem, Int Ed.

[4] Wang L, Chen S, Xu T, Taghizadeh K, Wishnok J S, Zhou X, You D, Deng Z, Dedon P C (2007). Nat Chem Biol.

[5] Kellner S, DeMott M S, Cheng C P, Russell B S, Cao B, You D, Dedon P C (2017). Nat Chem Biol.

[6] Wu Y, Tang Y, Dong X, Zheng Y Y, Haruehanroengra P, Mao S, Lin Q, Sheng J (2020). ACS Chem Biol.

[7] Züst R, Cervantes-Barragan L, Habjan M, Maier R, Neuman B W, Ziebuhr J, Szretter K J, Baker S C, Barchet W, Diamond M S (2011). Nat Immunol.

[8] Motorin Y, Marchand V (2018). Genes.

[9] Elliott B A, Ho H-T, Ranganathan S V, Vangaveti S, Ilkayeva O, Abou Assi H, Choi A K, Agris P F, Holley C L (2019). Nat Commun.

[10] Dimitrova D G, Teysset L, Carré C (2019). Genes.

[11] Freier S M, Altmann K-H (1997). Nucleic Acids Res.

[12] Manoharan M (1999). Biochim Biophys Acta, Gene Struct Expression.

[13] Manoharan M, Rajeev K G, Crooke S T (2008). Utilizing Chemistry to Harness RNA Interference Pathways for Therapeutics: Chemically Modified siRNAs and Antagomirs. Antisense Drug Technology.

[14] Ni S, Yao H, Wang L, Lu J, Jiang F, Lu A, Zhang G (2017). Int J Mol Sci.

[15] Egli M, Manoharan M (2019). Acc Chem Res.

[16] Yamasaki K, Akutsu Y, Yamasaki T, Miyagishi M, Kubota T (2020). Nucleic Acids Res.

[17] Hyjek-Składanowska M, Vickers T A, Napiórkowska A, Anderson B A, Tanowitz M, Crooke S T, Liang X-h, Seth P P, Nowotny M (2020). J Am Chem Soc.

[18] Shen X, Corey D R (2018). Nucleic Acids Res.

[19] Crooke S T, Witztum J L, Bennett C F, Baker B F (2018). Cell Metab.

[20] Roberts T C, Langer R, Wood M J A (2020). Nat Rev Drug Discovery.

[21] Egli M, Manoharan M Nucleic Acids Res.

[22] Furukawa Y, Kobayashi K, Kanai Y, Honjo M (1965). Chem Pharm Bull.

[23] Eckstein F (1966). J Am Chem Soc.

[24] Codington J F, Doerr I, Van Praag D, Bendich A, Fox J J (1961). J Am Chem Soc.

[25] Westheimer F (1987). Science.

[26] Kamerlin S C L, Sharma P K, Prasad R B, Warshel A (2013). Q Rev Biophys.

[27] Rich A (2003). Nat Struct Mol Biol.

[28] Egli M, Zhang S (2018). Sugar Pucker and Nucleic Acid Structure. The Excitement of Discovery: Selected Papers of Alexander Rich: A Tribute to Alexander Rich.

[29] Tamura M, Holbrook S R (2002). J Mol Biol.

[30] Butcher S E, Pyle A M (2011). Acc Chem Res.

[31] Denning E J, MacKerell A D (2012). J Am Chem Soc.

[32] Darré L, Ivani I, Dans P D, Gómez H, Hospital A, Orozco M (2016). J Am Chem Soc.

[33] Shi Y (2017). Nat Rev Mol Cell Biol.

[34] Fica S M, Nagai K (2017). Nat Struct Mol Biol.

[35] Peebles C L, Perlman P S, Mecklenburg K L, Petrillo M L, Tabor J H, Jarrell K A, Cheng H-L (1986). Cell.

[36] Qin P Z, Pyle A M (1998). Curr Opin Struct Biol.

[37] Cech T (1987). Science.

[38] Emilsson G M, Nakamura S, Roth A, Breaker R R (2003). RNA.

[39] O'Rourke S M, Scott W G (2018). Prog Mol Biol Transl Sci.

[40] Egli M, Lubini P, Pallan P S (2007). Chem Soc Rev.

[41] Egli M, Minasov G, Tereshko V, Pallan P S, Teplova M, Inamati G B, Lesnik E A, Owens S R, Ross B S, Prakash T P (2005). Biochemistry.

[42] Deleavey G F, Damha M J (2012). Chem Biol.

[43] Seth P P, Swayze E E, Hanessian S (2014). Unnatural Nucleoside Analogs for Antisense Therapy. Natural Products in Medicinal Chemistry.

[44] Campbell M A, Wengel J (2011). Chem Soc Rev.

[45] Kotikam V, Rozners E (2020). Acc Chem Res.

[46] Wan W B, Migawa M T, Vasquez G, Murray H M, Nichols J G, Gaus H, Berdeja A, Lee S, Hart C E, Lima W F (2014). Nucleic Acids Res.

[47] Iwamoto N, Butler D C D, Svrzikapa N, Mohapatra S, Zlatev I, Sah D W Y, Meena, Standley S M, Lu G, Apponi L H (2017). Nat Biotechnol.

[48] Schöning K-U, Scholz P, Guntha S, Wu X, Krishnamurthy R, Eschenmoser A (2000). Science.

[49] Maier T, Przylas I, Strater N, Herdewijn P, Saenger W (2005). J Am Chem Soc.

[50] Kumar P, Degaonkar R, Guenther D C, Abramov M, Schepers G, Capobianco M, Jiang Y, Harp J, Kaittanis C, Janas M M (2020). Nucleic Acids Res.

[51] Maiti M, Maiti M, Knies C, Dumbre S, Lescrinier E, Rosemeyer H, Ceulemans A, Herdewijn P (2015). Nucleic Acids Res.

[52] Ovaere M, Herdewijn P, Van Meervelt L (2011). Chem – Eur J.

[53] Moulton H M, Moulton J D (2017). Morpholino Oligomers.

[54] Schlegel M K, Foster D J, Kel’in A V, Zlatev I, Bisbe A, Jayaraman M, Lackey J G, Rajeev K G, Charissé K, Harp J (2017). J Am Chem Soc.

[55] Anosova I, Kowal E A, Dunn M R, Chaput J C, Van Horn W D, Egli M (2016). Nucleic Acids Res.

[56] Devine K G, Jheeta S (2020). Life.

[57] Eriksson M, Nielsen P E (1996). Q Rev Biophys.

[58] Teplova M, Minasov G, Tereshko V, Inamati G B, Cook P D, Manoharan M, Egli M (1999). Nat Struct Biol.

[59] Crooke S T, Baker B F, Kwoh T J, Cheng W, Schulz D J, Xia S, Salgado N, Bui H-H, Hart C E, Burel S A (2016). Mol Ther.

[60] Koch T (2013). Curr Phys Chem.

[61] Manoharan M, Akinc A, Pandey R K, Qin J, Hadwiger P, John M, Mills K, Charisse K, Maier M A, Nechev L (2011). Angew Chem, Int Ed.

[62] Pallan P S, Greene E M, Jicman P A, Pandey R K, Manoharan M, Rozners E, Egli M (2011). Nucleic Acids Res.

[63] Patra A, Paolillo M, Charisse K, Manoharan M, Rozners E, Egli M (2012). Angew Chem, Int Ed.

[64] Gryaznov S, Chen J-K (1994). J Am Chem Soc.

[65] Gryaznov S M, Lloyd D H, Chen J K, Schultz R G, DeDionisio L A, Ratmeyer L, Wilson W D (1995). Proc Natl Acad Sci U S A.

[66] Chen J-K, Schultz R G, Lioyd D H, Gryaznov S M (1995). Nucleic Acids Res.

[67] Skorski T, Perrotti D, Nieborowska-Skorska M, Gryaznov S, Calabretta B (1997). Proc Natl Acad Sci U S A.

[68] Heidenreich O, Gryaznov S, Nerenberg M (1997). Nucleic Acids Res.

[69] Giovannangeli C, Diviacco S, Labrousse V, Gryaznov S, Charneau P, Helene C (1997). Proc Natl Acad Sci U S A.

[70] Rigl C T, Lloyd D H, Tsou D S, Gryaznov S M, Wilson W D (1997). Biochemistry.

[71] Tereshko V, Gryaznov S, Egli M (1998). J Am Chem Soc.

[72] Escude C, Giovannangeli C, Sun J S, Lloyd D H, Chen J K, Gryaznov S M, Garestier T, Helene C (1996). Proc Natl Acad Sci U S A.

[73] Mutisya D, Hardcastle T, Cheruiyot S K, Pallan P S, Kennedy S D, Egli M, Kelley M L, van Brabant Smith A, Rozners E (2017). Nucleic Acids Res.

[74] Wilds C J, Minasov G, Natt F, von Matt P, Altmann K-H, Egli M (2001). Nucleosides, Nucleotides Nucleic Acids.

[75] Mutisya D, Selvam C, Lunstad B D, Pallan P S, Haas A, Leake D, Egli M, Rozners E (2014). Nucleic Acids Res.

[76] Idziak I, Just G, Damha M J, Giannaris P A (1993). Tetrahedron Lett.

[77] Rozners E, Katkevica D, Bizdena E, Strömberg R (2003). J Am Chem Soc.

[78] von Matt P, De Mesmaeker A, Pieles U, Zürcher W, Altmann K-H (1999). Tetrahedron Lett.

[79] Selvam C, Thomas S, Abbott J, Kennedy S D, Rozners E (2011). Angew Chem, Int Ed.

[80] Iwase R, Kurokawa R, Ueno J (2009). Nucleic Acids Symp Ser.

[81] Epple S, Thorpe C, Baker Y R, El-Sagheer A H, Brown T (2020). Chem Commun.

[82] Elkayam E, Kuhn C-D, Tocilj A, Haase A D, Greene E M, Hannon G J, Joshua-Tor L (2012). Cell.

[83] Hardcastle T, Novosjolova I, Kotikam V, Cheruiyot S K, Mutisya D, Kennedy S D, Egli M, Kelley M L, Smith A v B, Rozners E (2018). ACS Chem Biol.

[84] Beaton G, Brill W K-D, Grandas A, Ma Y-X, Nielsen J, Yau E, Caruthers M H (1991). Tetrahedron.

[85] Marshall W, Caruthers M (1993). Science.

[86] Iyer R P, Egan W, Regan J B, Beaucage S L (1990). J Am Chem Soc.

[87] Wickstrom E (2015). Adv Drug Delivery Rev.

[88] Clavé G, Reverte M, Vasseur J-J, Smietana M (2021). RSC Chem Biol.

[89] Pallan P S, Lybrand T P, Schlegel M K, Harp J M, Jahns H, Manoharan M, Egli M (2020). Biochemistry.

[90] Caruthers M H (1991). Acc Chem Res.

[91] Yang X, Sierant M, Janicka M, Peczek L, Martinez C, Hassell T, Li N, Li X, Wang T, Nawrot B (2012). ACS Chem Biol.

[92] Pallan P S, Yang X, Sierant M, Abeydeera N D, Hassell T, Martinez C, Janicka M, Nawrot B, Egli M (2014). RSC Adv.

[93] Wu S Y, Yang X, Gharpure K M, Hatakeyama H, Egli M, McGuire M H, Nagaraja A S, Miyake T M, Rupaimoole R, Pecot C V (2014). Nat Commun.

[94] Egli M, Lybrand T P (2019). J Am Chem Soc.

[95] Abeydeera N D, Egli M, Cox N, Mercier K, Conde J N, Pallan P S, Mizurini D M, Sierant M, Hibti F-E, Hassell T (2016). Nucleic Acids Res.

[96] Ueda N, Kawabata T, Takemoto K (1971). J Heterocycl Chem.

[97] Seita T, Yamauchi K, Kinoshita M, Imoto M (1972). Makromol Chem.

[98] Acevedo O L, Andrews R S (1996). Tetrahedron Lett.

[99] Zhang L, Peritz A, Meggers E (2005). J Am Chem Soc.

[100] Chen J J, Cai X, Szostak J W (2009). J Am Chem Soc.

[101] Schlegel M K, Peritz A E, Kittigowittana K, Zhang L, Meggers E (2007). ChemBioChem.

[102] Tsai C-H, Chen J, Szostak J W (2007). Proc Natl Acad Sci U S A.

[103] Meggers E, Zhang L (2010). Acc Chem Res.

[104] Nielsen P, Dreiøe L H, Wengel J (1995). Bioorg Med Chem.

[105] Schlegel M K, Essen L-O, Meggers E (2008). J Am Chem Soc.

[106] Johnson A T, Schlegel M K, Meggers E, Essen L-O, Wiest O (2011). J Org Chem.

[107] Schlegel M K, Essen L-O, Meggers E (2010). Chem Commun.

[108] Inoue H, Hayase Y, Imura A, Iwai S, Miura K, Ohtsuka E (1987). Nucleic Acids Res.

[109] Ross B S, Springer R H, Tortorici Z, Dimock S (1997). Nucleosides Nucleotides.

[110] Beigelman L, Haeberli P, Sweedler D, Karpeisky A (2000). Tetrahedron.

[111] Roy S K, Tang J-y (2000). Org Process Res Dev.

[112] Cummins L L, Owens S R, Risen L M, Lesnik E A, Freier S M, McGee D, Guinosso C J, Cook P D (1995). Nucleic Acids Res.

[113] Inoue H, Hayase Y, Iwai S, Ohtsuka E (1987). FEBS Lett.

[114] Lubini P, Zürcher W, Egli M (1994). Chem Biol.

[115] Lesnik E A, Guinosso C J, Kawasaki A M, Sasmor H, Zounes M, Cummins L L, Ecker D J, Cook P D, Freier S M (1993). Biochemistry.

[116] Martin P (1995). Helv Chim Acta.

[117] Legorburu U, Reese C B, Song Q (1999). Tetrahedron.

[118] Chow S, Wen K, Sanghvi Y S, Theodorakis E A (2003). Nucleosides, Nucleotides Nucleic Acids.

[119] Altmann K-H, Fabbro D, Dean N M, Geiger T, Monia B P, Müllert M, Nicklin P (1996). Biochem Soc Trans.

[120] Tereshko V, Portmann S, Tay E C, Martin P, Natt F, Altmann K-H, Egli M (1998). Biochemistry.

[121] Prakash T P (2011). Chem Biodiversity.

[122] Stephenson M L, Zamecnik P C (1978). Proc Natl Acad Sci U S A.

[123] Barabino S M L, Blencowe B J, Ryder U, Sproat B S, Lamond A I (1990). Cell.

[124] Iribarren A M, Sproat B S, Neuner P, Sulston I, Ryder U, Lamond A I (1990). Proc Natl Acad Sci U S A.

[125] Boiziau C, Larrouy B, Sproat B S, Toulmè J-J (1995). Nucleic Acids Res.

[126] Obika S, Nanbu D, Hari Y, Morio K-i, In Y, Ishida T, Imanishi T (1997). Tetrahedron Lett.

[127] Singh S K, Koshkin A A, Wengel J, Nielsen P (1998). Chem Commun.

[128] Koshkin A A, Singh S K, Nielsen P, Rajwanshi V K, Kumar R, Meldgaard M, Olsen C E, Wengel J (1998). Tetrahedron.

[129] Nielsen C B, Singh S K, Wengel J, Jacobsen J P (1999). J Biomol Struct Dyn.

[130] Braasch D A, Corey D R (2001). Chem Biol.

[131] Obika S, Nanbu D, Hari Y, Andoh J-i, Morio K-i, Doi T, Imanishi T (1998). Tetrahedron Lett.

[132] Petersen M, Wengel J (2003). Trends Biotechnol.

[133] Singh S K, Wengel J (1998). Chem Commun.

[134] Wengel J (1999). Acc Chem Res.

[135] Bondensgaard K, Petersen M, Singh S K, Rajwanshi V K, Kumar R, Wengel J, Jacobsen J P (2000). Chem – Eur J.

[136] Petersen M, Bondensgaard K, Wengel J, Jacobsen J P (2002). J Am Chem Soc.

[137] Schmidt K S, Borkowski S, Kurreck J, Stephens A W, Bald R, Hecht M, Friebe M, Dinkelborg L, Erdmann V A (2004). Nucleic Acids Res.

[138] Kaur H, Babu B R, Maiti S (2007). Chem Rev.

[139] Fluiter K, ten Asbroek A L M A, de Wissel M B, Jakobs M E, Wissenbach M, Olsson H, Olsen O, Oerum H, Baas F (2003). Nucleic Acids Res.

[140] Grünweller A, Wyszko E, Bieber B, Jahnel R, Erdmann V A, Kurreck J (2003). Nucleic Acids Res.

[141] Schubert S, Gül D C, Grunert H-P, Zeichhardt H, Erdmann V A, Kurreck J (2003). Nucleic Acids Res.

[142] Kuespert S, Heydn R, Peters S, Wirkert E, Meyer A-L, Siebörger M, Johannesen S, Aigner L, Bogdahn U, Bruun T-H (2020). Int J Mol Sci.

[143] van Ravesteyn T W, Dekker M, Fish A, Sixma T K, Wolters A, Dekker R J, te Riele H P J (2016). Proc Natl Acad Sci U S A.

[144] Ju E, Li T, Liu Z, da Silva S R, Wei S, Zhang X, Wang X, Gao S-J (2020). ACS Nano.

[145] Fluiter K, Frieden M, Vreijling J, Rosenbohm C, De Wissel M B, Christensen S M, Koch T, Ørum H, Baas F (2005). ChemBioChem.

[146] Rajwanshi V K, Håkansson A E, Dahl B M, Wengel J (1999). Chem Commun.

[147] Nielsen K M E, Petersen M, Håkansson A E, Wengel J, Jacobsen J P (2002). Chem – Eur J.

[148] Jørgensen A S, Hansen L H, Vester B, Wengel J (2014). Bioorg Med Chem Lett.

[149] Raguraman P, Wang T, Ma L, Jørgensen P T, Wengel J, Veedu R N (2020). Int J Mol Sci.

[150] Singh S K, Kumar R, Wengel J (1998). J Org Chem.

[151] Astakhova I K, Wengel J (2014). Acc Chem Res.

[152] Rosenbohm C, Christensen S M, Sørensen M D, Pedersen D S, Larsen L-E, Wengel J, Koch T (2003). Org Biomol Chem.

[153] Sørensen M D, Petersen M, Wengel J (2003). Chem Commun.

[154] Hrdlicka P J, Babu B R, Sørensen M D, Wengel J (2004). Chem Commun.

[155] Valeur E, Bradley M (2009). Chem Soc Rev.

[156] Baxter E W, Reitz A B (2004). Reductive Aminations of Carbonyl Compounds with Borohydride and Borane Reducing Agents. Organic Reactions.

[157] Obika S, Onoda M, Morita K, Andoh J-i, Koizumi M, Imanishi T (2001). Chem Commun.

[158] Morihiro K, Kodama T, Kentefu, Moai Y, Veedu R N, Obika S (2013). Angew Chem, Int Ed.

[159] Moai Y, Hiroaki H, Obika S, Kodama T (2020). Nucleosides, Nucleotides Nucleic Acids.

[160] Xu J, Liu Y, Dupouy C, Chattopadhyaya J (2009). J Org Chem.

[161] Egli M, Minasov G, Teplova M, Kumar R, Wengel J (2001). Chem Commun.

[162] Seth P P, Siwkowski A, Allerson C R, Vasquez G, Lee S, Prakash T P, Kinberger G, Migawa M T, Gaus H, Bhat B (2008). Nucleic Acids Symp Ser.

[163] Seth P P, Vasquez G, Allerson C A, Berdeja A, Gaus H, Kinberger G A, Prakash T P, Migawa M T, Bhat B, Swayze E E (2010). J Org Chem.

[164] Blade H, Bradley D, Diorazio L, Evans T, Hayter B R, Howell G P (2015). J Org Chem.

[165] Hong D, Kurzrock R, Kim Y, Woessner R, Younes A, Nemunaitis J, Fowler N, Zhou T, Schmidt J, Jo M (2015). Sci Transl Med.

[166] Barman J, Gurav D, Oommen O P, Varghese O P (2015). RSC Adv.

[167] Mitsuoka Y, Aoyama H, Kugimiya A, Fujimura Y, Yamamoto T, Waki R, Wada F, Tahara S, Sawamura M, Noda M (2016). Org Biomol Chem.

[168] Vejlegaard K, Wegeberg C, McKee V, Wengel J (2018). Org Biomol Chem.

[169] Seth P P, Allerson C R, Berdeja A, Siwkowski A, Pallan P S, Gaus H, Prakash T P, Watt A T, Egli M, Swayze E E (2010). J Am Chem Soc.

[170] Ito Y, Tsutsui N, Osawa T, Hari Y (2019). J Org Chem.

[171] Kumar P, El-Sagheer A H, Truong L, Brown T (2017). Chem Commun.

[172] Sharma V K, Singh S K, Krishnamurthy P M, Alterman J F, Haraszti R A, Khvorova A, Prasad A K, Watts J K (2017). Chem Commun.

[173] Seley-Radtke K L, Yates M K (2018). Antiviral Res.

[174] Fox J J (1969). Pure Appl Chem.

[175] Glaudemans C P J, Fletcher H G (1963). J Org Chem.

[176] Tann C H, Brodfuehrer P R, Brundidge S P, Sapino C, Howell H G (1985). J Org Chem.

[177] Howell H G, Brodfuehrer P R, Brundidge S P, Benigni D A, Sapino C (1988). J Org Chem.

[178] Wilds C J, Damha M J (2000). Nucleic Acids Res.

[179] Elzagheid M I, Viazovkina E, Damha M J (2002). Curr Protoc Nucleic Acid Chem.

[180] Elzagheid M I, Viazovkina E, Damha M J (2003). Nucleosides, Nucleotides Nucleic Acids.

[181] Damha M J, Usman N, Ogilvie K K (1989). Can J Chem.

[182] Giannaris P A, Damha M J (1994). Can J Chem.

[183] Noronha A M, Wilds C J, Lok C-N, Viazovkina K, Arion D, Parniak M A, Damha M J (2000). Biochemistry.

[184] Viazovkina E, Mangos M M, Elzagheid M I, Damha M J (2002). Curr Protoc Nucleic Acid Chem.

[185] Kawasaki A M, Casper M D, Freier S M, Lesnik E A, Zounes M C, Cummins L L, Gonzalez C, Cook P D (1993). J Med Chem.

[186] Damha M J, Wilds C J, Noronha A, Brukner I, Borkow G, Arion D, Parniak M A (1998). J Am Chem Soc.

[187] Lok C-N, Viazovkina E, Min K-L, Nagy E, Wilds C J, Damha M J, Parniak M A (2002). Biochemistry.

[188] Kalota A, Karabon L, Swider C R, Viazovkina E, Elzagheid M, Damha M J, Gewirtz A M (2006). Nucleic Acids Res.

[189] Berger I, Tereshko V, Ikeda H, Marquez V E, Egli M (1998). Nucleic Acids Res.

[190] Li F, Sarkhel S, Wilds C J, Wawrzak Z, Prakash T P, Manoharan M, Egli M (2006). Biochemistry.

[191] Trempe J-F, Wilds C J, Denisov A Y, Pon R T, Damha M J, Gehring K (2001). J Am Chem Soc.

[192] Denisov A Yu, Noronha A M, Wilds C J, Trempe J-F, Pon R T, Gehring K, Damha M J (2001). Nucleic Acids Res.

[193] Salazar M, Fedoroff O Y, Miller J M, Ribeiro N S, Reid B R (1993). Biochemistry.

[194] Fedoroff O Y, Salazar M, Reid B R (1993). J Mol Biol.

[195] Watts J K, Martín-Pintado N, Gómez-Pinto I, Schwartzentruber J, Portella G, Orozco M, González C, Damha M J (2010). Nucleic Acids Res.

[196] Martín-Pintado N, Yahyaee-Anzahaee M, Campos-Olivas R, Noronha A M, Wilds C J, Damha M J, González C (2012). Nucleic Acids Res.

[197] Martin-Pintado N, Deleavey G F, Portella G, Campos-Olivas R, Orozco M, Damha M J, González C (2013). Angew Chem, Int Ed.

[198] Dowler T, Bergeron D, Tedeschi A-L, Paquet L, Ferrari N, Damha M J (2006). Nucleic Acids Res.

[199] Kel′in A V, Zlatev I, Harp J, Jayaraman M, Bisbe A, O′Shea J, Taneja N, Manoharan R M, Khan S, Charisse K (2016). J Org Chem.

[200] Harp J M, Guenther D C, Bisbe A, Perkins L, Matsuda S, Bommineni G R, Zlatev I, Foster D J, Taneja N, Charisse K (2018). Nucleic Acids Res.

[201] Liboska R, Snášel J, Barvík I, Buděšínský M, Pohl R, Točík Z, Páv O, Rejman D, Novák P, Rosenberg I (2011). Org Biomol Chem.

[202] Petrová M, Páv O, Buděšínský M, Zborníková E, Novák P, Rosenbergová Š, Pačes O, Liboska R, Dvořáková I, Šimák O (2015). Org Lett.

[203] Jenkins I D, Verheyden J P H, Moffatt J G (1976). J Am Chem Soc.

[204] Owen G R, Verheyden J P H, Moffatt J G (1976). J Org Chem.

[205] Thomas S O, Singleton V L, Lowery J A, Sharpe R W, Pruess L M, Porter J N, Mowat J H, Bohonos N (1956). Antibiot Annu.

[206] Martínez-Montero S, Deleavey G F, Kulkarni A, Martín-Pintado N, Lindovska P, Thomson M, González C, Götte M, Damha M J (2014). J Org Chem.

[207] Martínez-Montero S, Deleavey G F, Martín-Pintado N, Fakhoury J F, González C, Damha M J (2015). ACS Chem Biol.

[208] Martínez-Montero S, Deleavey G F, Dierker-Viik A, Lindovska P, Ilina T, Portella G, Orozco M, Parniak M A, González C, Damha M J (2015). J Org Chem.

[209] Malek-Adamian E, Patrascu M B, Jana S K, Martínez-Montero S, Moitessier N, Damha M J (2018). J Org Chem.

[210] Malek-Adamian E, Guenther D C, Matsuda S, Martínez-Montero S, Zlatev I, Harp J, Burai Patrascu M, Foster D J, Fakhoury J, Perkins L (2017). J Am Chem Soc.

[211] Li Q, Chen J, Trajkovski M, Zhou Y, Fan C, Lu K, Tang P, Su X, Plavec J, Xi Z (2020). J Am Chem Soc.

[212] Gore K R, Nawale G N, Harikrishna S, Chittoor V G, Pandey S K, Höbartner C, Patankar S, Pradeepkumar P I (2012). J Org Chem.

[213] Koizumi K, Maeda Y, Kano T, Yoshida H, Sakamoto T, Yamagishi K, Ueno Y (2018). Bioorg Med Chem.

[214] Kano T, Katsuragi Y, Maeda Y, Ueno Y (2018). Bioorg Med Chem.

[215] Nawale G N, Bahadorikhalili S, Sengupta P, Kadekar S, Chatterjee S, Varghese O P (2019). Chem Commun.

[216] Herdewijn P (2010). Chem Biodiversity.

[217] Aerschot Van A, Verheggen I, Hendrix C, Herdewijn P (1995). Angew Chem, Int Ed Engl.

[218] Hendrix C, Rosemeyer H, Verheggen I, Van Aerschot A, Seela F, Herdewijn P (1997). Chem – Eur J.

[219] Hossain N, Wroblowski B, Van Aerschot A, Rozenski J, De Bruyn A, Herdewijn P (1998). J Org Chem.

[220] Allart B, Khan K, Rosemeyer H, Schepers G, Hendrix C, Rothenbacher K, Seela F, Van Aerschot A, Herdewijn P (1999). Chem – Eur J.

[221] Egli M, Pallan P S, Allerson C R, Prakash T P, Berdeja A, Yu J, Lee S, Watt A, Gaus H, Bhat B (2011). J Am Chem Soc.

[222] Winter H D, Lescrinier E, Aerschot A V, Herdewijn P (1998). J Am Chem Soc.

[223] Vandermeeren M, Préveral S, Janssens S, Geysen J, Saison-Behmoaras E, Van Aerschot A, Herdewijn P (2000). Biochem Pharmacol.

[224] Allerson C R, Sioufi N, Jarres R, Prakash T P, Naik N, Berdeja A, Wanders L, Griffey R H, Swayze E E, Bhat B (2005). J Med Chem.

[225] Davis S, Propp S, Freier S M, Jones L E, Serra M J, Kinberger G, Bhat B, Swayze E E, Bennett C F, Esau C (2009). Nucleic Acids Res.

[226] Prakash T P, Yu J, Kinberger G A, Low A, Jackson M, Rigo F, Swayze E E, Seth P P (2018). Bioorg Med Chem Lett.

[227] Pallan P S, Yu J, Allerson C R, Swayze E E, Seth P, Egli M (2012). Biochemistry.

[228] Seth P P, Allerson C R, Østergaard M E, Swayze E E (2012). Bioorg Med Chem Lett.

[229] De S, Jabgunde A M, Patil R S, De Jonghe S, Beigelman L, Herdewijn P (2018). J Org Chem.

[230] Jabgunde A M, Patil R S, De S, Lescrinier E, De Jonghe S, Beigelman L, Herdewijn P (2019). Tetrahedron.

[231] Schweitzer B A, Kool E T (1994). J Org Chem.

[232] Moran S, Ren R X-F, Rumney S, Kool E T (1997). J Am Chem Soc.

[233] Khakshoor O, Wheeler S E, Houk K N, Kool E T (2012). J Am Chem Soc.

[234] Kellinger M W, Ulrich S, Chong J, Kool E T, Wang D (2012). J Am Chem Soc.

[235] Xia J, Noronha A, Toudjarska I, Li F, Akinc A, Braich R, Frank-Kamenetsky M, Rajeev K G, Egli M, Manoharan M (2006). ACS Chem Biol.

[236] Ulrich S, Kool E T (2011). Biochemistry.

[237] Li F, Pallan P S, Maier M A, Rajeev K G, Mathieu S L, Kreutz C, Fan Y, Sanghvi J, Micura R, Rozners E (2007). Nucleic Acids Res.

[238] Egli M, Pallan P S (2007). Annu Rev Biophys Biomol Struct.

[239] Egli M (2012). Acc Chem Res.

[240] Pallan P S, Egli M (2009). J Am Chem Soc.

